# p21-activated kinase 4 controls the aggregation of α-synuclein by reducing the monomeric and aggregated forms of α-synuclein: involvement of the E3 ubiquitin ligase NEDD4-1

**DOI:** 10.1038/s41419-022-05030-1

**Published:** 2022-06-30

**Authors:** So-Yoon Won, Jung-Jin Park, Soon-Tae You, Jong-A Hyeun, Hyong-Kyu Kim, Byung Kwan Jin, Catriona McLean, Eun-Young Shin, Eung-Gook Kim

**Affiliations:** 1 Department of Biochemistry & Molecular Biology, Department of Neuroscience, Neurodegeneration Control Research Center, School of Medicine, Kyung Hee University, Seoul, 02447 South Korea; 2 Department of Biochemistry and Medical Research Center, Chungbuk National University College of Medicine, Cheongju, 28644 South Korea; 3 Department of Neurosurgery, the Catholic University of Korea, St. Vincent’s Hospital, Suwon, Gyeonggi-do 16247 South Korea; 4 Department of Medicine and Microbiology, Chungbuk National University College of Medicine, Cheongju, 28644 South Korea; 5 Department of Pathology, The Alfred Hospital, Melbourne, VIC 3004 Australia

**Keywords:** Parkinson's disease, Cell death in the nervous system

## Abstract

Aggregation of misfolded alpha-synuclein (α-synuclein) is a central player in the pathogenesis of neurodegenerative diseases. Therefore, the regulatory mechanism underlying α-synuclein aggregation has been intensively studied in Parkinson’s disease (PD) but remains poorly understood. Here, we report p21-activated kinase 4 (PAK4) as a key regulator of α-synuclein aggregation. Immunohistochemical analysis of human PD brain tissues revealed an inverse correlation between PAK4 activity and α-synuclein aggregation. To investigate their causal relationship, we performed loss-of-function and gain-of-function studies using conditional PAK4 depletion in nigral dopaminergic neurons and the introduction of lentivirus expressing a constitutively active form of PAK4 (caPAK4; PAK4^S445N/S474E^), respectively. For therapeutic relevance in the latter setup, we injected lentivirus into the striatum following the development of motor impairment and analyzed the effects 6 weeks later. In the loss-of-function study, Cre-driven PAK4 depletion in dopaminergic neurons enhanced α-synuclein aggregation, intracytoplasmic Lewy body-like inclusions and Lewy-like neurites, and reduced dopamine levels in PAK4^DAT-CreER^ mice compared to controls. Conversely, caPAK4 reduced α-synuclein aggregation, as assessed by a marked decrease in both proteinase K-resistant and Triton X100-insoluble forms of α-synuclein in the AAV-α-synuclein-induced PD model. Mechanistically, PAK4 specifically interacted with the NEDD4-1 E3 ligase, whose pharmacological inhibition and knockdown suppressed the PAK4-mediated downregulation of α-synuclein. Collectively, these results provide new insights into the pathogenesis of PD and suggest PAK4-based gene therapy as a potential disease-modifying therapy in PD.

## Introduction

Parkinson’s disease (PD) is an age-dependent disorder characterized by a progressive degeneration of dopamine neurons in the substantia nigra (SN) pars compacta, leading to a decrease in dopamine levels in the striatum [1–3]. When diagnosed, most PD patients present a loss of >60% of dopamine neurons [4, 5], which critically requires disease-modifying therapy (DMT) for their management [6–8]. However, an effective DMT that would stop the ongoing degeneration of dopamine neurons and maintain dopamine levels is currently lacking [9, 10]. To overcome this limit, a deeper understanding of the molecular mechanisms underlying the pathogenesis of PD is needed.

Aggregation of alpha-synuclein (α-synuclein) plays a central role in the pathogenesis of neurodegenerative diseases [11, 12]. The pathological hallmark of PD is defined by the presence of Lewy bodies (LBs) in cell bodies and Lewy neurites, whose main component is aggregated α-synuclein [13, 14]. These α-synuclein inclusions are found in both familial and sporadic cases of PD [15, 16]; thus, α-synuclein aggregation is considered a common denominator of PD pathogenesis. Of note, α-synuclein phosphorylation at S129 has been observed in the brains of PD patients and transgenic models of PD [17–19]. Although it is not clear whether this phosphorylation mediates α-synuclein aggregation, it has been frequently used as an index of aggregation.

Aggregated α-synuclein induces cellular dysfunction through multiple routes, including mitochondrial dysfunction, chronic inflammation, synaptic dysfunction, nuclear dysfunction, inter-organelle defects, ER/Golgi dysfunction, and autophagy/lysosomal dysfunction [20–27]. A number of studies have focused on understanding ways to prevent α-synuclein aggregation or remove α-synuclein oligomers/fibrillar aggregates to develop therapeutic strategies for PD [5, 28]. Aggregates of α-synuclein in neuronal cells are cleared in a number of ways, including ubiquitination-dependent degradation systems. Previous work has shown that several E3 ligases [such as seven in absentia homolog (SIAH), carboxyl terminus of Hsp70-interacting protein (CHIP), and neuronal precursor cell-expressed, developmentally downregulated gene 4 (NEDD4)] are implicated in the ubiquitination of α-synuclein [29–31]. NEDD4 is a HECT (homologous to E6AP C-terminus)-domain E3 ligase that uniquely functions by trafficking α-synuclein for degradation through the endosome–lysosome pathway [32]. NEDD4 mediates Lys-63-linked ubiquitination of α-synuclein, which is known to target membrane proteins to lysosomes [33]. Through this trafficking of α-synuclein, NEDD4 prevents α-synuclein accumulation and is thus protective against α-synuclein toxicity [34]. It is clear that NEDD4 plays a unique role in the turnover of α-synuclein; however, its upstream regulator(s) remain poorly understood.

p21-activated kinase 4 (PAK4) is a member of the PAK family of serine/threonine kinases, which regulate a wide range of cellular functions including cell adhesion, migration, proliferation, and survival [35, 36]. Thus, dysregulation of its expression and activity contributes to the development of diverse pathological conditions [35–37]. We previously reported that levels of phosphorylated PAK4 (pPAK4^S474^), an index of PAK4 activity, were reduced in patients with PD compared to age-matched controls [37]. Danzer et al. reported that α-synuclein oligomers inhibit the kinase activity of PAK4 as assessed by the level of its autophosphorylation in vitro [38]. This in vitro finding was further supported in α-synuclein (A30P) transgenic mice that showed a decrease in the phosphorylation levels of LIMK, a substrate of PAK4 [38]. Pretreatment with the constitutively active form of PAK4 (caPAK4; PAK4^S445N/S474E^) prevented the degeneration of dopamine neurons in an α-synuclein-induced model of PD [37]. However, whether caPAK4 affects α-synuclein aggregation in this event has not yet been determined.

In this study, we sought to address the role of PAK4 in α-synuclein aggregation in the pathogenesis of PD. We performed both loss-of-function and gain-of-function studies. Our data reveal PAK4 as a key regulator of α-synuclein aggregation and suggest the involvement of NEDD4 E3 ligase as a downstream effector of PAK4 in this event.

## Results

### PAK4 activity is inversely correlated with α-synuclein aggregation

**Fig. 1:**
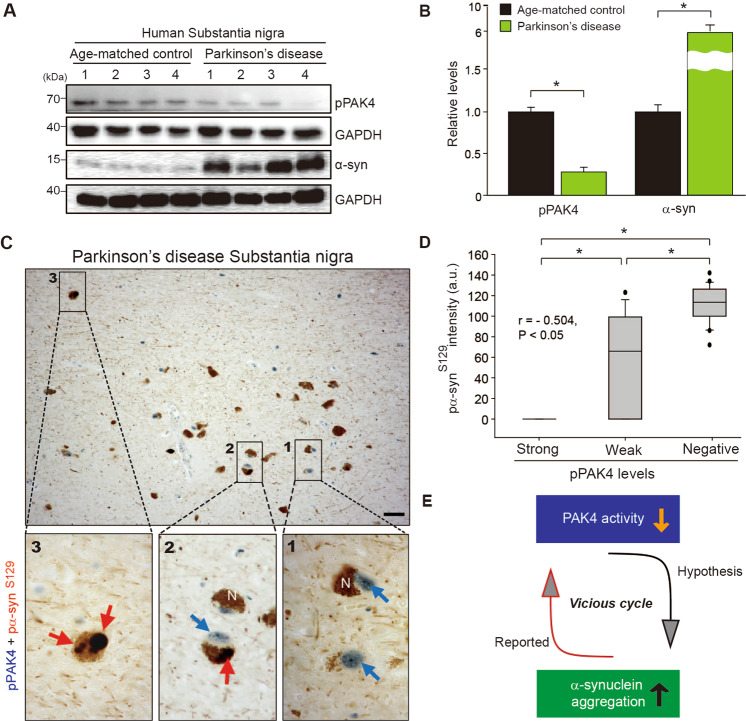
Inverse correlations between pPAK4 levels and α-synuclein aggregation in the substantia nigra of postmortem brain tissue from patients with PD. **A** Immunoblotting of SN lysates for pPAK4 and α-synuclein. **B** Quantification of the blot in **A** normalized to GAPDH. Age-matched controls and PD patients (*n* = 4 for each group). **C** Costaining for pPAK4 and pα-syn^S129^. Scale bars, 25 µm. Boxed areas in **C** are shown at higher magnification. *N* neuromelanin. pPAK4, blue arrow; α-synuclein, red arrow. **D** Spearman correlation analysis for an inverse correlation between pPAK4 and pα-syn^S129^ levels in human PD tissues. PD patients, *n* = 7; neuromelanin-positive cells, *n* = 63. *r* = −0.504, *P* < 0.05. **E** Model for the relationship between PAK4 activity and α-synuclein aggregation. The data are presented as the mean ± SEM. *P < 0.05. Unpaired Student’s *t* test (**B**) or two-way ANOVA (**D**) was used for statistical analysis followed by Tukey’s multiple comparisons test.

Considering a neuroprotective role for PAK4 in α-syn PD models [37], we asked whether PAK4 activity may affect α-synuclein aggregation. We first examined the status of pPAK4, an index of PAK4 activity, and α-synuclein separately in the SN of brain tissues from PD patients and age-matched controls (Supplemental Fig. 1 and Supplemental Table S1). pPAK4 stained strongly in neuromelanin-positive neurons from age-matched controls, whereas pPAK4 signals were rarely detected in PD brain tissues (Supplemental Fig. 1A–C), which is consistent with the previous result [37]. In contrast, PD brain tissues displayed strong immunoreactivity of α-synuclein (Supplemental Fig. 1D–F) and pα-synuclein^S129^ (pα-syn^S129^), an index of α-synuclein aggregates (Supplemental Fig. 1G–I), as previously reported [26, 39, 40]. Collectively, these results suggested a possible link between PAK4 activity and the accumulation of α-synuclein. Therefore, we examined their correlations at the level of tissues and single cells by immunoblotting and immunostaining, respectively. Immunoblotting revealed a marked reduction in pPAK4 levels in the SN tissues from PD patients, whereas α-synuclein accumulated at higher levels (Fig. 1A, B). To further examine their correlation at the cellular level, we performed double immunostaining. In the postmortem PD brain tissue analysis, strong pPAK4-positive cells displayed no signals of pα-syn^S129^ (Fig. 1C, inset 1, D). Weakly pPAK4-stained neuromelanin-positive cells showed some heterogeneity for pα-syn^S129^ (Fig. 1C, inset 2, D). In contrast, the pPAK4 signal was mostly negative in the strongly pα-syn^S129^-positive cells (Fig. 1C, inset 3, D); thus, pPAK4 levels and α-synuclein aggregation apparently showed an inverse correlation (*r* = −0.504, *P* < 0.05). Together, these results led us to develop a hypothesis that PAK4 may be implicated in the aggregation of α-synuclein under pathophysiological conditions; decreased PAK4 activity in PD may contribute to α-synuclein aggregation, thus forming a vicious cycle (Fig. 1E).

### Ablation of nigral dopaminergic PAK4 promotes α-synuclein aggregation in the SN of the mouse brain

**Fig. 2:**
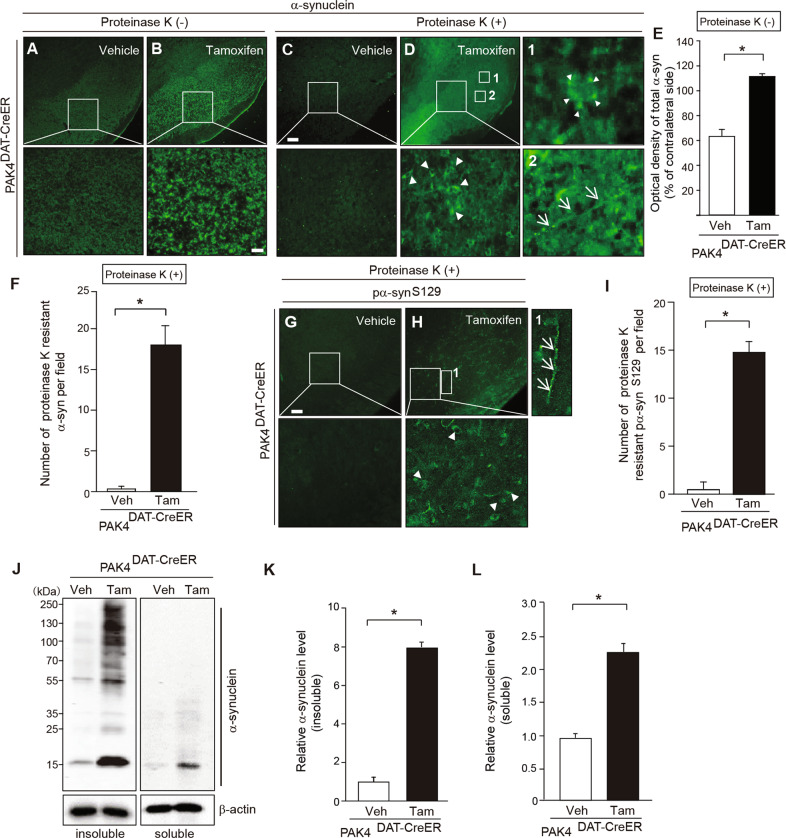
PAK4 ablation results in increased α-synuclein aggregation in the mouse SN. **A**–**D** Representative images of α-synuclein immunostaining in SN from PAK4^DAT-CreER^ mice treated with vehicle (**A**, **C**) or tamoxifen (**B**, **D**). Large boxed areas (**A**–**D**) are shown at higher magnification in the respective bottom panel. Boxed areas 1 and 2 (**D**) are enlarged to reveal granular deposits (arrowheads) and neurites (arrows). Scale bar, 50 µm. **E** Quantification of the intensity of total α-synuclein staining (**A**, **B**) (*n* = 5 mice per group). **F** Quantification of the number of proteinase K-resistant α-synuclein inclusions (*n* = 5 mice per group). **G**, **H** Representative images of pα-syn^S129^ immunostaining in SN from PAK4^DAT-CreER^ mice treated with vehicle (**G**) or tamoxifen (**H**). Large boxed areas (**G**, **H**) and boxed area 1 (**H**) are enlarged to reveal granular deposits (arrowheads) and neurites (arrows), respectively. Scale bar, 50 µm. **I** Quantification of the number of proteinase K-resistant pα-syn^S129^ inclusions in SN (*n* = 5 for each group). **J** Representative immunoblots for α-synuclein in Triton X-100-insoluble and soluble fractions of SN from vehicle (Veh)- and tamoxifen (Tam)-treated PAK4^DAT-CreER^ mice. **K**, **L** Quantification of the density of α-synuclein normalized to β-actin in Triton X-100-insoluble (**K**) and soluble (**L**) fractions (*n* = 4 for each group). The data are presented as the mean ± SEM. *P < 0.01. Unpaired Student’s *t* test.

To test our hypothesis that PAK4 may regulate α-synuclein aggregation in dopamine neurons, we ablated the PAK4 gene using the Cre/loxP system in dopamine neurons by crossing PAK4^flox/flox^ mice homozygous for the PAK4 floxed allele [41] with transgenic mice harboring a tamoxifen-inducible DAT-Cre (DAT^Cre/+^) gene [42]. PCR analysis confirmed the generation of PAK4^DAT-CreER^ mice (Supplemental Fig. 2A). When tamoxifen or vehicle was administered to these mice, as depicted in Supplemental Fig. 2B, PAK4 levels were significantly reduced in the SN tissues of tamoxifen, but not in vehicle-treated mice (Supplemental Fig. 2C, D). By double immunofluorescence analyses, we confirmed a specific decrease in PAK4 expression in TH-positive dopamine neurons from tamoxifen-injected PAK4^DAT-CreER^ mice (Supplemental Fig. 2E, F). The accumulation of insoluble higher molecular weight species of α-synuclein is a pathological hallmark of PD [43, 44]. To examine the effect of PAK4 on α-synuclein aggregation, we performed immunohistochemical analyses on tissue sections after treatment with proteinase K, which allows selective visualization of proteinase K-resistant aggregates. Vehicle-injected PAK4^DAT-CreER^ mice showed no detectible inclusions, as monitored by staining for α-synuclein (Fig. 2A, C) and its phosphorylated form (pα-syn^S129^) (Fig. 2G, I). In contrast, tamoxifen-injected PAK4^DAT-CreER^ mice displayed increased staining for total α-synuclein (Fig. 2B, E) and numerous inclusions of proteinase K-resistant α-synuclein (Fig. 2D, F) and pα-syn^S129^ (Fig. 2H, I). Among these α-synuclein inclusions, some were round lamellated cytoplasmic inclusions (Fig. 2D, inset 1) and Lewy neurite-like aggregates along axons (Fig. 2D, inset 2 and H, inset 1). To further evaluate α-synuclein aggregation in PAK4-deficient mice, we fractionated soluble and insoluble α-synuclein by sequential extraction of the SN tissues and performed immunoblotting. In the insoluble fraction, tamoxifen-injected PAK4^DAT-CreER^ mice showed a marked accumulation of higher molecular weight species and monomeric forms of α-synuclein compared to vehicle-injected controls (Fig. 2J, left panel, K). In the soluble fraction, a significant amount of α-synuclein monomers was also detected in tamoxifen-treated mice, whereas higher molecular forms were barely detectible (Fig. 2J, right panel, L). These results clearly indicate that PAK4 ablation promotes the aggregation of α-synuclein and the accumulation of monomeric α-synuclein in SN.

### Constitutively active PAK4 reduces α-synuclein aggregation in A53T animal models of PD

**Fig. 3:**
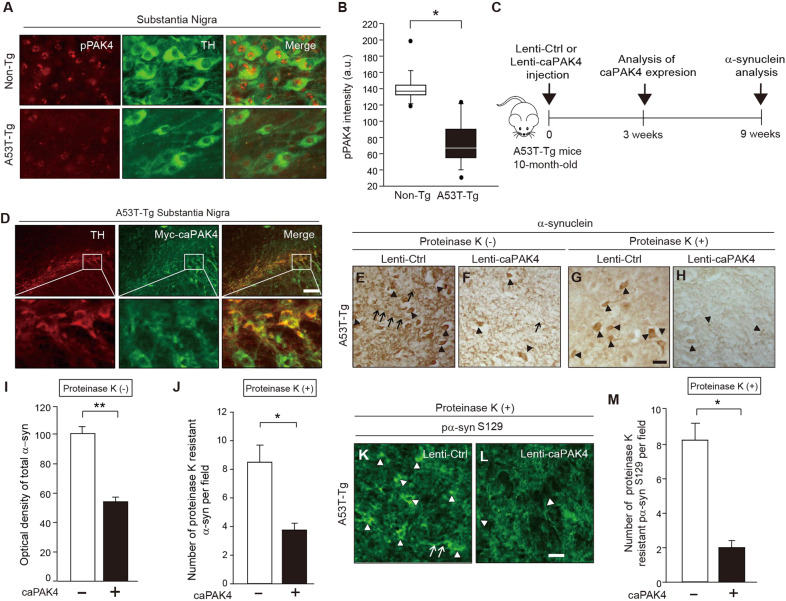
caPAK4 reduces α-synuclein aggregates in the A53T PD model. **A** Double immunostaining for pPAK4 (red) and TH (green). Scale bar, 100 µm. **B** Quantification of pPAK4 staining intensity in TH-positive dopamine neurons (a.u., arbitrary units) (*n* = 5 for each group). **C** Experimental scheme. **D** Expression of caPAK4 (GFP; green) in TH-positive dopamine neurons of A53T-Tg mice. **E**–**H** Representative images of α-synuclein immunostaining in the SN of A53T-Tg mice injected with lenti-Ctrl or lenti-caPAK4. Scale bar, 100 µm. **I** Quantification of the intensity of total α-synuclein (**E**, **F**) in SN (*n* = 5 for each group). **J** Quantification of the number of proteinase K-resistant α-synuclein inclusions (**G**, **H**) in SN (*n* = 5 for each group). **K**, **L** Representative images of pα-syn^S129^ immunostaining in the SN of A53T-Tg mice injected with lenti-Ctrl or lenti-caPAK4. Scale bar, 100 µm. **M** Quantification of the number of pα-syn^S129^-positive cells in SN. Arrowheads and arrows indicate granular deposits and neurites, respectively. The data are presented as the mean ± SEM. *P < 0.01, **P < 0.01. Unpaired Student’s *t* test.

Considering the effect of PAK4 depletion on the disruption of α-synuclein physiology, we wondered whether caPAK4 suppresses the aggregation of α-synuclein in pathological conditions. Therefore, we investigated the effects of caPAK4 in A53T-transgenic (Tg) mice that express a mutant form of human α-synuclein (hα-syn) and thus progressively develop α-synuclein aggregation [45, 46]. To evaluate the status of PAK4 activity, we assessed pPAK4 levels in the dopamine neurons of A53T-Tg mice. The intensity of pPAK4 was apparently reduced in TH-positive dopamine neurons of A53T-Tg mice compared to non-Tg mice, suggesting decreased PAK4 activity (Fig. 3A, B). Next, we determined whether caPAK4 lessens the burden of hα-syn in the SN of A53T-Tg mice. Lenti-caPAK4 was stereotactically injected into the striatum of 10-month-old A53T–Tg mice (Fig. 3C). At 3 weeks post lenti-caPAK4, the expression of caPAK4 was confirmed in TH-positive dopamine neurons (Fig. 3D). At 9 weeks after injection of the lenti-Ctrl A53T-Tg mice, α-synuclein aggregates (Fig. 3E, arrowheads), Lewy neurite-like aggregates (Fig. 3E, arrows), and a number of large proteinase K-resistant α-synuclein aggregates (Fig. 3G, arrowheads) were observed. In contrast, caPAK4 expression significantly reduced both the optical density of α-synuclein (Fig. 3F, I) and proteinase K-resistant α-synuclein (Fig. 3H, J). Consistent with this result, lenti-caPAK4 greatly reduced pα-syn^S129^, an index of α-synuclein aggregation, in the SN of A53T-Tg mice compared with lenti-Ctrl injected A53T-tg mice (Fig. 3K–M). Together, these results indicate that PAK4 is capable of reducing α-synuclein aggregation in disease conditions.

### Constitutively active PAK4 reduces α-synuclein aggregation in α-synuclein-induced animal models of PD

**Fig. 4:**
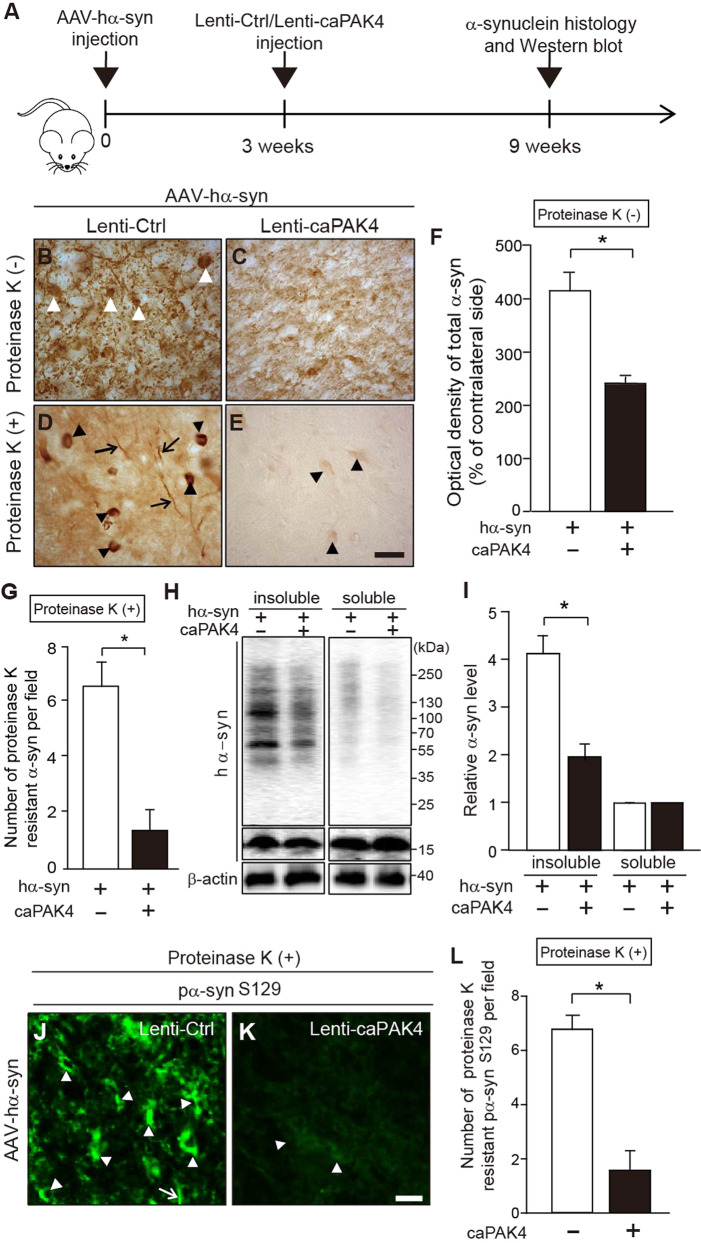
caPAK4 reduces α-synuclein aggregates in the AAV-hα-syn-induced PD model. **A** Experimental scheme. **B**–**E** Representative images of α-synuclein immunostaining in the SN of lenti-Ctrl- or lenti-caPAK4-injected rats post AAV-hα-syn. Arrowheads, α-synuclein aggregates; arrow, Lewy-like neurites. Scale bar, 100 µm. **F** Quantification of the intensity of total α-synuclein (**B**, **C**) in SN (*n* = 5 mice per group). **G** Quantification of the number of proteinase K-resistant α-synuclein inclusions (**D**, **E**) in the SN (*n* = 5 mice per group). **H** Representative immunoblots for α-synuclein in Triton X-100-insoluble and soluble fractions of the SN from lenti-Ctrl- or lenti-caPAK4-injected rats post AAV-hα-syn. **I** Quantification of the density of α-synuclein in Triton X-100-insoluble and soluble fractions normalized to β-actin (*n* = 5 for each group). **J**, **K** Representative images of pα-syn^S129^ immunostaining in the SN of lenti-Ctrl- or lenti-caPAK4-injected rats post AAV-hα-syn. Scale bar, 100 µm. **L** Quantification of the number of pα-syn^S129^-positive cells in the SN. Arrowheads, granular deposits; arrows, neurites. The data are presented as the mean ± SEM. *P < 0.01. Unpaired Student’s *t* test.

We further investigated the effects of caPAK4 in a rat model of PD unilaterally overexpressing hα-syn, as outlined in Fig. 4A. First, we confirmed adeno-associated virus (AAV)- and lentivirus-mediated expression of hα-syn and caPAK4, respectively. AAV-hα-syn expressing GFP as a reporter was stereotactically injected into SN (Supplemental Fig. 3A, B). Overexpression of hα-syn was detected in the cytoplasm of TH-positive neurons in the rat SN 3 weeks after injection of AAV-hα-syn (Supplemental Fig. 3C). Then, lenti-caPAK4 or lenti-Ctrl was injected into the striatum of these rats at 3 weeks post AAV-hα-syn injection (Supplemental Fig. 4A, B). Two weeks after stereotactic injection, lenti-caPAK4 was transduced mainly into TH-positive dopamine neurons in SN (Supplemental Fig. 4C). To evaluate whether caPAK4 affects the aggregation of α-synuclein, we performed immunohistochemical analysis 9 weeks after AAV-hα-syn injection (Fig. 4B–G). AAV-hα-syn/lenti-Ctrl-injected rats showed α-synuclein aggregates of various sizes and shapes (Fig. 4B, white arrowheads) and a number of large proteinase K-resistant α-synuclein aggregates (Fig. 4D, black arrowheads). Lewy neurite-like aggregates were also frequently detected in these rats (Fig. 4D, arrows). However, caPAK4 expression post AAV-hα-syn significantly decreased the optical density of α-synuclein (Fig. 4C, F) and almost eliminated proteinase K-resistant α-synuclein (Fig. 4E, G) compared with controls. Next, for immunoblotting analysis, we separated SN tissue extracts into soluble and insoluble fractions 9 weeks after AAV-hα-syn injection (Fig. 4A). AAV-hα-syn/lenti-Ctrl-injected rats showed an accumulation of high molecular weight species of α-synuclein in the insoluble fraction but weakly detectible levels in the soluble fraction (Fig. 4H, I). Of note, lenti-caPAK4 treatment reduced α-synuclein levels in the insoluble fraction by >50% compared to the control condition (Fig. 4H, I). Consistent with this result, lenti-caPAK4 greatly reduced pα-syn^S129^ in the SN of AAV-hα-syn-injected rats compared with the control (Fig. 4J–L).

### Constitutively active PAK4 rescues degenerating dopamine neurons in α-synuclein-induced animal models of PD

**Fig. 5:**
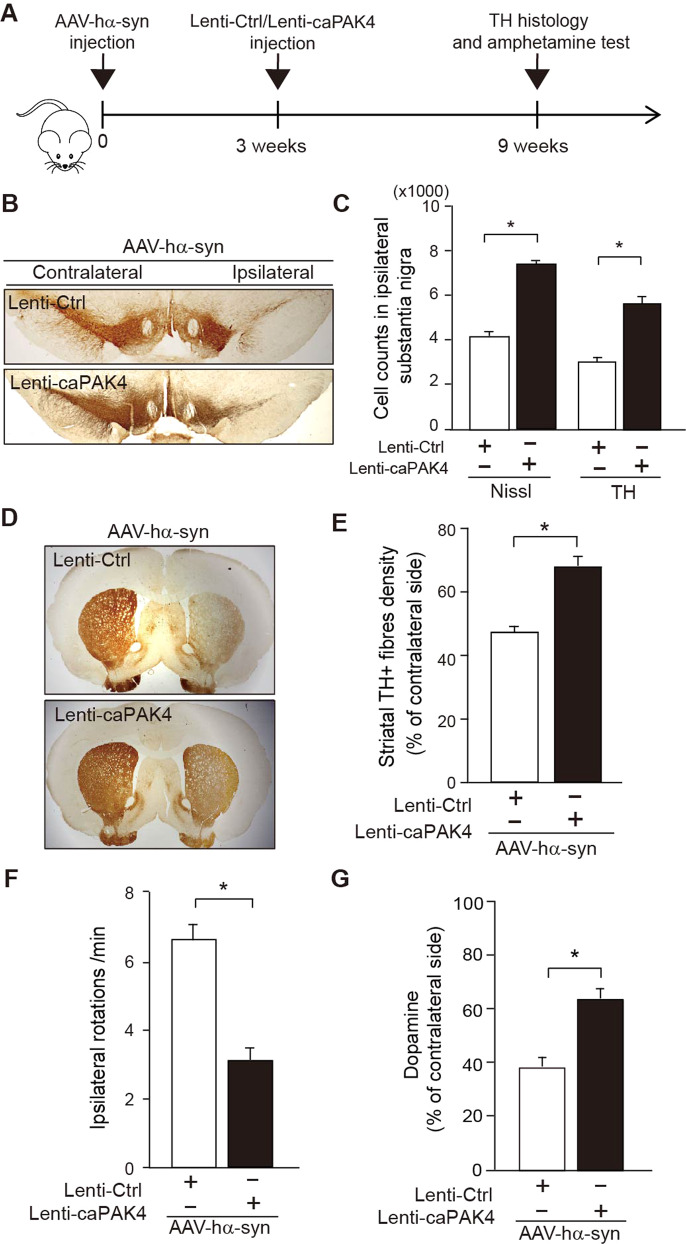
Post-treatment with caPAK4 shows a therapeutic effect in the α-synuclein PD model. **A** Experimental scheme. **B** Representative images of TH immunostaining in the SN from lenti-Ctrl- or lenti-caPAK4-injected rats post AAV-hα-syn. **C** Stereological counting of TH-positive and Nissl-stained dopamine neurons (*n* = 5 for each group). **D** Representative images of TH immunostaining in the rat striatum. **E** Quantification of striatal TH-positive fiber density (*n* = 5 for each group). **F** Quantification of cumulative amphetamine-induced ipsilateral rotations (*n* = 5 for each group). **G** Quantification of dopamine levels in the ipsilateral striatum from lenti-Ctrl- or lenti-caPAK4-injected rats post AAV-hα-syn (*n* = 5 for each group). The data are presented as the mean ± SEM. *P < 0.01. Unpaired Student’s *t* test.

Given that PAK4 controls α-synuclein aggregation, we expected that PAK4 would show PD-modifying potential. For this purpose, we designed an experimental scheme to represent a clinically relevant setup for therapeutic intervention (Fig. 5A). Amphetamine-induced ipsilateral rotation was analyzed at 3 weeks after the AAV-hα-syn injection. Rats that exhibited ipsilateral rotations, indicative of an effective lesion, were randomly selected for treatment with the lenti-Ctrl or lenti-caPAK4 in the striatum for 2 weeks (Supplemental Fig. 4A, B). The number of TH-positive and Nissl-stained dopamine neurons as assessed by stereology in the SNpc and the density of TH-positive fibers in the striatum are significantly higher in the AAV-hα-syn-lesioned rats treated with lenti-caPAK4, compared with the lenti-Ctrl-treated rats (Fig. 5B–E). Lenti-caPAK4 also significantly attenuated amphetamine-induced rotations indicative of behavioral rescue and elevated dopamine levels (Fig. 5F, G). Taken together, these results strongly suggest that caPAK4 may modify the progression of PD (Supplemental Fig. 5).

### Constitutively active PAK4 reduces α-synuclein levels via NEDD4-1

**Fig. 6:**
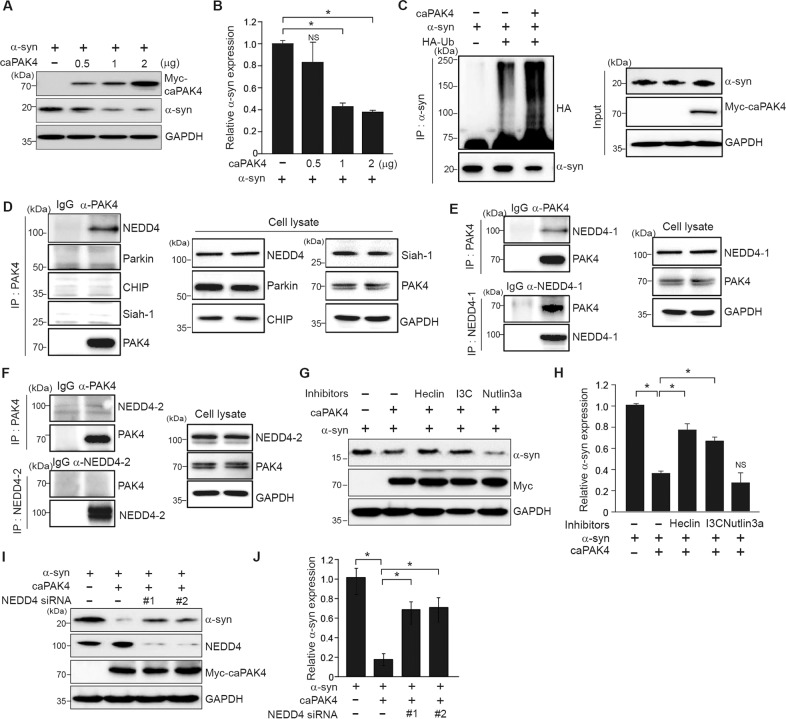
NEDD4-1 is required for caPAK4-induced α-synuclein degradation in SHSY-5Y cells. **A** Immunoblotting for α-synuclein in SH-SY5Y cells transfected with plasmids encoding vector alone or human Myc-caPAK4. **B** Quantification of α-synuclein intensity in blot **A** normalized to GAPDH. **C** In vitro ubiquitination of α-synuclein. **D** Immunoprecipitation (IP) and immunoblotting for the indicated E3 ligases. **E**, **F** Immunoprecipitation (IP) and immunoblotting. SH-SY5Y cell lysates were immunoprecipitated with anti-PAK4 or anti-NEDD4-1 (**E**)/anti-NEDD4-2 (**F**) antibodies followed by immunoblotting for PAK4, NEDD4-1, and GAPDH. **G** Immunoblotting for α-synuclein in SH-SY5Y cells following incubation with the indicated E3 ligase inhibitors. **H** Quantification of the α-synuclein signal in the blot **E** normalized to GAPDH. **I** Immunoblotting for α-synuclein in SH-SY5Y cells transfected with NEDD4-1 siRNAs. **J** Quantification of α-synuclein levels in the blot shown (**I**) normalized to GAPDH. The data are presented as the mean ± SEM. *P < 0.01. Two-way ANOVA (**B**, **H**, **J**) was used for statistical analysis followed by Tukey’s multiple comparisons test.

We sought to understand the mechanism underlying PAK4-regulated α-synuclein aggregation in a cell (SH-SY5Y)-based assay. First, we determined whether caPAK4 expression reduced α-synuclein levels in vitro. With increasing concentrations of transfected caPAK4 in SH-SY5Y cells, α-synuclein levels gradually decreased (Fig. 6A, B). It is well established that α-synuclein is subjected to ubiquitination-dependent degradation [29]. Therefore, we tested whether PAK4 may affect the ubiquitination of α-synuclein. Forced expression of caPAK4 markedly increased the levels of ubiquitinated α-synuclein (Fig. 6C). Next, to search for E3 ligase that ubiquitinates α-synuclein, we performed immunoprecipitation. PAK4 interacted with NEDD4-1 (NEDD4) but not with other E3 ligases, Parkin, CHIP, and Siah-1 (Fig. 6D). We further examined the binding difference between two close isoforms: NEDD4-1 and NEDD4-2. Of note, PAK4 interacted with NEDD4-1 but not NEDD4-2, indicating its binding specificity (Fig. 6E, F). These results suggested a possible involvement of NEDD4-1 E3 ligase in PAK4-regulated α-synuclein aggregation. We tested this idea by employing two different approaches: a pharmacologic and genetic approach. The caPAK4-mediated reduction in α-synuclein levels was inhibited by heclin, an inhibitor of E3 ligases containing the HECT domain, and indole-3-carbinol (I3C), a NEDD4-1 inhibitor, but not by the MDM2 inhibitor nutlin-3a (Fig. 6G, H). When NEDD4-1 was knocked down using two different siRNAs against NEDD4-1, the α-synuclein-reducing effect of PAK4 was inhibited (Fig. 6I, J). Taken together, NEDD4-1 functioned downstream of PAK4 in the degradation of α-synuclein.

### PAK4 enhances the association of NEDD4-1 with α-synuclein

**Fig. 7:**
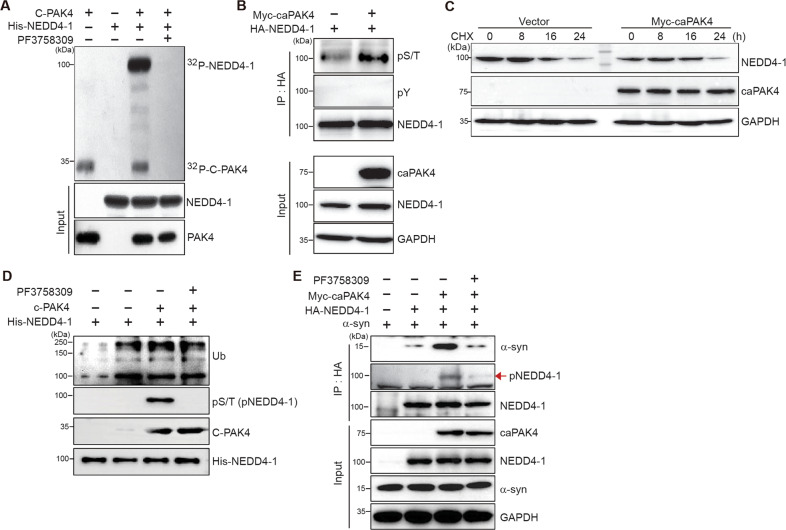
PAK4 enhances the association of NEDD4-1 with α-synuclein through phosphorylation of NEDD4-1. **A** In vitro kinase assay for PAK4-mediated phosphorylation of NEDD4-1 with vehicle (control) or PF3758309 (10 μM). C-PAK4, C-terminal kinase domain of PAK4. **B** Immunoblotting for NEDD4-1 with anti-phospho-serine/threonine (α-pS/T) and anti-phospho-tyrosine (α-pY) antibodies in vector- or myc-caPAK4–transfected cells. **C** Immunoblotting for NEDD4-1 in vector- or myc-caPAK4–transfected cells over 24 h following treatment with cycloheximide (CHX; 20 μg/ml). **D** In vitro ubiquitination assay to assess the catalytic activity of NEDD4-1 with vehicle (control) or PF3758309 (10 μM). **E** Immunoblotting after immunoprecipitation with anti-HA (NEDD4-1) with vehicle (control) or PF3758309 (10 μM). Each blot is representative of at least two experiments.

We further explored the mechanism of NEDD4-1 regulation by PAK4. Because of their interaction, we hypothesized that PAK4 likely phosphorylates NEDD4-1. To test this possibility, we performed in vitro kinase assay by using the recombinant C-terminal kinase domain of PAK4 and NEDD4-1 (Fig. 7A). NEDD4-1 was markedly phosphorylated by PAK4, with PAK4 auto-phosphorylation also detectable. Pretreatment with the PAK4 inhibitor PF3758309 completely blocked NEDD4-1 phosphorylation, indicating that it was specific (last lane). In 293T cells expressing NEDD4-1 (HA-tagged) and PAK4 (Myc-tagged), phosphorylated NEDD4-1 was detected by anti-phospho-serine/threonine antibody but not anti-phospho-tyrosine antibody (Fig. 7B). To assess whether PAK4-mediated NEDD4-1 phosphorylation stabilizes NEDD4-1, degradation of endogenous NEDD4-1 was chased following treatment with cycloheximide in the absence or presence of caPAK4. NEDD4-1 stability was not considerably affected by caPAK4 (Fig. 7C). It is conceivable that PAK4-mediated phosphorylation of NEDD4-1 may affect its ubiquitination activity. To verify this assumption, we measured its self-ubiquitination activity. The self-ubiquitination of NEDD4-1 occurred at similar levels regardless of PAK4 activity, which was assessed by phosphorylation of NEDD4-1 (Fig. 7D). Consistent with this result, pretreatment with PF3758309 induced no obvious changes. Finally, we examined the effect of NEDD4-1 phosphorylation on the interaction between NEDD4-1 and α-synuclein. 293T cells were transfected with plasmids encoding Myc-caPAK4, HA-NEDD4-1, and α-synuclein, and their association was analyzed by immunoprecipitation with anti-HA (NEDD4-1) followed by immunoblotting for α-synuclein. As reported previously [33], an association between NEDD4-1 and α-synuclein was detected (Fig. 7E, 2^nd^ lane). caPAK4 notably increased their association (3^rd^ lane), and this increase was almost completely blocked by PF3758309 (4^th^ lane). Thus, PAK4-mediated phosphorylation of NEDD4-1 enhanced its association with α-synuclein. This association may accelerate the ubiquitination of α-synuclein and its subsequent endosomal targeting for lysosomal degradation.

## Discussion

Aging has long been known to be a risk factor for PD, for which the major pathogenic mechanism is α-synuclein aggregation that occurs in both the familial and sporadic forms of PD. We previously reported that PAK4 activity declines with aging [37], suggesting that decreased PAK4 activity might contribute to an increased risk of developing PD. However, how decreased PAK4 activity might constitute an aging-related risk factor remains unclear. We hypothesized that PAK4 might be implicated in α-synuclein aggregation. Notably, the findings from the present study, in which we specifically depleted dopaminergic PAK4 and delivered caPAK4 by lentivirus, support the role of PAK4 as an aging-related risk factor. Consistent with this evidence, analysis of brain tissues from patients with PD revealed an inverse correlation between PAK4 activity and α-synuclein aggregation. Thus, maintaining PAK4 activity in older individuals may prevent the build-up of α-synuclein aggregates and could represent a therapeutic modality for the management of PD.

Autophagy/ubiquitin-dependent proteolytic systems are involved in the clearance of α-synuclein aggregates [23, 47]. Polo-like kinase 2 (PLK2) has been reported to degrade phosphorylated a-synuclein^S129^ via the autophagy-lysosomal pathway [39]. Interestingly, polyubiquitination of PLK2 itself is a signal that can be recognized by the autophagy machinery. However, the specific E3 ligase responsible for this ubiquitination is unclear. PINK1 also interacts with α-synuclein and induces degradation of α-synuclein via autophagy [48]. PAK4 greatly increased the ubiquitination of α-synuclein, which resulted in its degradation. NEDD4-1 functioned downstream of PAK4 in this event. NEDD4-1 extends the ubiquitin chain at Lys-63 on α-synuclein [33], suggesting that PAK4-induced α-synuclein degradation may occur via the endosome-lysosomal pathway [32]. PAK4 phosphorylated NEDD4-1, which increased its association with α-synuclein and perhaps subsequent Lys-63-mediated polyubiquitination for its endosomal targeting and lysosomal degradation. Currently, the NEDD4-1 residue(s) phosphorylated by PAK4 is unknown. Considering the importance of NEDD4-1 phosphorylation in its catalytic activity [49], our findings provide an additional phosphorylation-dependent regulatory mechanism by which PAK4 regulates the degradation of α-synuclein. Identification of novel phospho-site(s) in NEDD4-1 and the correlation to its target ubiquitination may improve our understanding of its function in healthy and diseased states. NEDD4-1 also extends Lys-63-ubiquitin chains to RTP801, a pro-apoptotic protein, and causes its degradation [50]. In accordance, reduced levels of NEDD4-1 but elevated levels of RTP801 were observed in nigral neurons from patients with PD [51]. Recent evidence highlights aberrant endolysosomal sorting as a pathogenic mechanism in PD [50, 52–54]. Thus, the PAK4-NEDD4-1 pathway may represent a novel target for therapeutic intervention in PD. Understanding the detailed mechanism for PAK4-induced clearance of α-synuclein may hold promise for other synucleopathies.

Our previous work showed that PAK4 plays a neuroprotective role via the CRTC1-CREB pathway [37]. This study provides another potential route, i.e., the NEDD4-1-dependent pathway. NEDD4-2 has been reported as a CREB target gene in hepatocytes [54]. In the liver, the CRTC2 isoform is a major coactivator of CREB, while CRTC1 is in the brain. Therefore, the expression of NEDD4-1 may be under the control of the CRTC1-CREB pathway in the brain. Together, it is plausible that the CRTC1-CREB pathway and NEDD4-1-dependent clearance of α-synuclein may play a synergistic role in the PAK4-mediated protection of dopaminergic neurons.

Given that PAK4 is protective against degeneration of dopaminergic neurons and that its activity declines with aging [37], decreased PAK4 activity may somehow facilitate α-synuclein aggregation and contribute to an increased risk of developing PD. Indeed, depleting PAK4 in nigral dopaminergic neurons resulted in the accumulation of aggregated forms of α-synuclein, cytoplasmic Lewy-like inclusions, and neurites. Together, these findings led us to develop a model in which PAK4 activity may regulate α-synuclein homeostasis. When PAK4 activity is sufficient to maintain α-synuclein levels below the threshold of aggregation, reduced levels of α-synuclein oligomers would not inhibit PAK4 activity. This physiological feedback loop between PAK4 activity and α-synuclein aggregation contributes to a healthy state. Conversely, higher levels of α-synuclein over the threshold of cellular clearance capacity, i.e., under conditions similar to PD, trigger the production of oligomeric α-synuclein, leading to a decrease in PAK4 activity (Fig. 1E, left arrow, red). Due to reduced PAK4 activity, the clearance of α-synuclein would be inefficient, further accelerating α-synuclein aggregation and thus forming a vicious cycle (Fig. 1E, right arrow, black). Thus, PAK4 activity at levels above a critical threshold appears to be crucial for preventing α-synuclein aggregation and keeping dopamine neurons healthy.

Although PAK4-deficient mice displayed α-synuclein aggregation, as illustrated by the presence of numerous proteinase K-resistant inclusions and Lewy-like neurites, we were not able to detect loss of nigral neurons. Accordingly, these mice exhibited no motor deficit during the observations up to 40 days. The development of motor symptoms may require several more months [55]. Although PD-inducing mutations in PAK4 have not been reported, their decreased levels in old ages may in part contribute to the pathogenesis. Even with these limitations, PAK4-deficient mice would be a good model for investigating the mechanism of α-synuclein aggregation.

In conclusion, our studies define a novel PAK4-NEDD4-1 pathway in the regulation of α-synuclein aggregation. This degradation route may offer opportunities for the development of new therapeutic strategies for PD.

## Materials and methods

### Antibodies and chemicals

The primary antibodies and chemicals used were as follows: rabbit anti-TH (Pel-Freez, Brown Deer), mouse anti-TH (MAB5280, Millipore), rabbit anti-phospho-PAK4 (S474) (#3241, Cell Signaling), rabbit anti-PAK4 (ab19007, Abcam), mouse anti-α-synuclein (SNCA) (ab80627, Abcam), mouse anti-α-synuclein (SNCA) (MAB5383, Abnova), rabbit anti-phospho-α-synuclein (S129) (SNCA) (ab51253, Abcam), mouse anti-phospho-α-synuclein (S129) (SNCA) (ab184674, Abcam), Anti-phospho-serine/threonine (ab17464, Abcam), anti-phospho-tyrosine (05-321, Upstate), rabbit anti-GAPDH (sc25778, Santa Cruz Biotechnology), mouse anti-Parkin (#4211, Cell Signaling), rabbit anti-Chip (#2080, Cell Signaling), goat anti-Siah-1 (ab2237, Abcam), rabbit anti-NEDD4-1 (#2740, Cell Signaling), rabbit anti-NEDD4-2 (ab46521), rabbit anti-GFP (sc8334, Santa Cruz Biotechnology), rabbit anti-Myc (sc789, Santa Cruz Biotechnology), rabbit anti-His (#2366S, Cell Signaling), and GST-conjugated horseradish peroxidase (HRP) (ab3416, Abcam), tamoxifen (T5648, Sigma-Aldrich), Heclin (SML1396, Sigma-Aldrich), indole-3-carbinol (I7256, Sigma-Aldrich), Nutlin3a (SML0580, Sigma-Aldrich), phosphatase inhibitor cocktails II (P5726, Sigma-Aldrich) and III (P0044, Sigma-Aldrich) and complete protease inhibitor mixture (#5871, Cell Signaling Technology, Danvers, MA, USA).

### siRNAs

Human NEDD4-1-specific siRNAs (4734-1 and 4734-3), human PAK4-specific siRNAs (#1: GAGUUGCCAGAGAAUGGUUdTdT, #2: CUGAGAUGAUAACUGUGAAdTdT), and negative control (SN-1003) were purchased from Bioneer (Daejeon, Korea).

### Human brain tissues

Human tissue was obtained from the Victoria Brain Bank (VBB). Experiments were performed in accordance with a protocol approved by the Ethics Review Committee of the Institutional Review Board of Chungbuk National University (approval number: CBNU-IRB-2011-T01). For immunohistochemistry, we analyzed samples from 7 PD patients and 7 age-matched controls (Supplemental Table S1). For immunoblotting, we analyzed samples from 4 PD patients and 4 age-matched controls (Supplemental. Table S1). PD was diagnosed using neuropathological criteria in conjunction with the clinical history [56]. The age-matched control tissue used was from patients without neuropathological evidence of PD or any other neurodegenerative disease or any other significant pathology upon neuropathologic examination, which included macroscopic and microscopic analysis.

### Animal models

All experimental procedures were performed in accordance with the guidelines of the Laboratory Animal Manual of the National Institutes of Health Guide to the Care and Use of Animals, which were approved by the Ethics Review Committee of Chungbuk National University for Animal Experiments (approval number: CBNUA-400-12-02). Rats were obtained from Taconic, Korea and were housed two to three per cage with ad libitum access to water and food during a 12-h light/dark cycle. Sprague-Dawley rats weighed 260–280 g at the time of surgery. Tamoxifen (1 mg/50 µl) or vehicle (sunflower oil) was injected intraperitoneally into PAK4-DAT-CreER mice twice a day for 7 days. A53T α-synuclein transgenic mice (004479-B6; C3-Tg (Prnp-SNCA*A53T)83Vle/J) were purchased from the Jackson Laboratory. All reasonable efforts were made to minimize animal suffering and to use the minimum number of animals necessary to perform statistically valid analyses.

### Viruses

Adeno-associated vector 5 (AAV5)-wild-type human α-synuclein and AAV5-GFP were kindly provided by the University of North Carolina VECTOR CORE. The AAV5 viruses used here are driven by a chicken beta-actin (CBA) promoter. Lenti-GFP and Lenti-caPAK4 were provided by Genenmed Inc. (Seoul, Korea). cDNA for active PAK4 (S445N/S474E) was subcloned into a lentiEZTM vector system.

### Delivery of virus by stereotaxic injection

Adenoviral vectors were stereotactically injected into the right SN. The injection site coordinates in the right SN were anteroposterior (AP) −5.3 mm, mediolateral (ML) −2.0 mm, and dorsoventricular (DV) −7.6 mm from bregma. In a single unilateral injection, 4 μl of AAV5 [AAV5-α-syn, 1.5 × 10^13^ viral genomes (vg)/ml] was delivered at a constant rate (0.25 μl/min) using a Hamilton RN syringe 33-gauge needle) with a neuroadapter attached to a syringe pump. After the injection, the needle was maintained in place for an additional 5 min to prevent retrograde flow along the needle track. Lentivirus particles can be transported to SN from the striatum by retrograde transport [56, 57]. Thus, the stereotactic injection site was the striatum [AP, 1.0 mm; ML, −3.0 mm; and DV, −5.0 mm from bregma]. In a single unilateral injection, 5 μl of lentivirus [caPAK4, 1.5 × 10^8^ transducing units (TU)/ml] was delivered at a constant rate (0.25 μl/min) using a Hamilton removable needle (RN) syringe (33-gauge needle) with a Neuro adapter attached to a syringe pump. A lentivirus expressing GFP alone was used as the control.

### Tissue preparation for immunostaining and immunoblotting analysis

For rat and mouse brains, animals were anesthetized with chloral hydrate (360 mg/kg, i.p.) at the indicated time points after injection and were perfused transcardially with a 0.9% saline solution containing 0.5% sodium nitrate and heparin (10 U/ml), which was followed by fresh cold 4% paraformaldehyde (PFA) fixative (pH 7.4). For histological studies, brains were frozen sectioned using a sliding microtome into 35 μm coronal sections after they were embedded in optimum cutting temperature (O.C.T) compound (Surgipath), and they were then collected into six separate series. Sections were stored free-floating in a cryopreservative medium at −20 °C. For biochemical studies, brains were removed, and SN was dissected under a dissection microscope. Blocks of tissue were frozen in dry ice and stored at −80 °C. Next, the tissue was processed for immunoblotting.

### Immunostaining of brain tissue

Paraffin-embedded human brain tissue slices [age-matched control and Parkinson’s disease subjects obtained from the VBB] were deparaffinized in xylene and subjected to citrate antigen retrieval prior to immunohistochemical analysis [58]. The tissue sections were washed in cold PBS for 15 min and incubated with a universal blocking solution in PBS (0.3% Triton X-100, 1% bovine serum albumin [BSA], 0.05% Tween 20, 0.1% cold fish gelatin, and 0.05% sodium azide) for 1 h at room temperature (RT). For light microscopy, brain tissues were incubated with a biotin-conjugated secondary antibody followed by streptavidin-conjugated HRP. Immunostaining was visualized by incubating the samples in a 0.1 M-PB solution containing DAB and 0.003% hydrogen peroxide. To coimmunostain for pPAK4 and pα-synS129, brain tissues were incubated with alkaline phosphatase-conjugated secondary antibodies (Vector Laboratories). Immunostaining was visualized using an Alkaline Phosphatase Substrate Kit III (blue) (Vector Laboratories). The animal brain tissue sections were washed in cold PBS for 15 min and incubated with a universal blocking solution in PBS for 1 h at RT. To detect aggregated α-syn, sections were pretreated with proteinase K (PK) for 8 min at 1 µg/ml as previously described [59] and immunostained with an antibody against total α-syn. For each case, three sections were analyzed using a digital video microscope (Olympus BX51, Tokyo, Japan) with the integrated Image-Pro Plus analysis system. Primary antibodies were diluted in Diluent antibody (Dako, S3022). For light microscopy, brain tissues were incubated with a biotin-conjugated secondary antibody followed by streptavidin-conjugated HRP (Vectastain ABC kit, Vector Laboratories). Immunostaining was visualized by incubating the samples in a 0.1 M-PB solution containing 0.05% diaminobenzidine-HCl and 0.003% hydrogen peroxide. For immunofluorescence staining, the tissue sections were incubated with Alexa 488- or Alexa 594-conjugated secondary antibodies. Fluorescence images were acquired by fluorescence microscopy (Nikon). All images were processed by Nikon software. For Nissl staining, SN tissues were mounted on gelatin-coated slides, dried for 1 h at RT, stained using 0.5% cresyl violet, dehydrated, cover-slipped, and then analyzed with bright-field microscopy (Nikon).

### Western blot

For the soluble fraction, tissues were homogenized in the following TX-soluble buffer (50 mM Tris [pH 8.0], 150 mM NaCl, 1% Triton-100) containing protease and phosphatase inhibitors; samples were centrifuged at 22,000 × *g* for 20 min, and the soluble supernatant was collected. The insoluble pellet was homogenized in TX-insoluble buffer containing 50 mM Tris [pH 8.0], 150 mM NaCl, 1% Triton X-100, and 2% SDS with protease inhibitor cocktail. The homogenates were centrifuged at 22,000 × *g* for 20 min. Immunoblotting of tissue was performed as described. The immunoblots were visualized using HRP-conjugated secondary antibodies against IgG and a chemiluminescent substrate.

### Immunoprecipitation

Cells were harvested in lysis buffer (50 mM HEPES, pH 7.5; 150 mM NaCl; 10% glycerol; 1% Triton X-100; 500 μM EDTA; 200 μM sodium pyruvate; 50 mM β-glycerophosphate) supplemented with protease inhibitors. Lysates were rotated at 4 °C for 1 h and then centrifuged at 14,000 rpm for 20 min. The supernatants were then immunoprecipitated using an anti-PAK4 antibody, anti-NEDD4-1 antibody, anti-NEDD4-2 at 4 °C for 18 h. Immunoprecipitates were collected by adding protein-G agarose and were washed five times with lysis buffer.

### Stereological cell counting

An unbiased stereological estimation of the total number of TH-positive neurons was conducted using the optical fractionator method using a bright-field microscope (Olympus Optical, BX43) and Stereo Investigator software (MBF Bioscience, Williston, VT). This unbiased stereological method of cell counting is not affected by either the reference volume (SNpc) or the size of the counted elements (neurons). The total number of neurons was estimated according to the Optical Fractionator Equation. More than 300 points covering all sections of each specimen were analyzed. All animals used in this study were coded, and a blind analysis was conducted for quantitative comparison. In addition, Nissl staining was performed to validate the neuron counts in SN, as previously described.

### Transfection

For transient transfection, a mixture of DNA or siRNA and Lipofectamine 2000 reagent (Invitrogen) was prepared according to the manufacturer’s instructions and incubated with cells for the indicated times.

### Measurement of dopamine levels

Dopamine was measured by using a dopamine ELISA Kit (BioVision, Inc., Milpitas, CA 95035). The brain tissues with ice-cold PBS (0.01 M, pH = 7.4) to remove excess hemolysis blood thoroughly. Tissue pieces should be weighed and then minced into small pieces, which will be homogenized in PBS (the volume depends on the weight of the tissue. In all, 9 ml PBS would be appropriate for 1 g of tissue. Some protease inhibitor is recommended to add into the PBS.) with a glass homogenizer on ice. To further break the cells, sonicate the suspension with an ultrasonic cell disrupter or subject it to freeze–thaw cycles. The homogenates are then centrifuged for 5 min at 5000 × *g* to retrieve the supernatant.

### Amphetamine test

Automated assessment of amphetamine-induced rotational behavior was performed using the TSE Video Mot2system (Germany) for 120 min after i.p. injection with 2.5 mg/kg D-amphetamine sulfate (Lipomed).

### Statistical and image analysis

The threshold was selected under Image/Adjust to achieve a desired range of intensity values for each experiment. The threshold setting was also used to exclude the background. After exclusion of the background, the selected area in the signal intensity range of the threshold was measured using the measurement option under the Analyze/Measure menu. The fluorescence intensity of α-synuclein within SN was measured using ImageJ analysis at different sections throughout the entire midbrain region of each animal. All experiments were performed blindly and in three independent experiments. For quantification of protein levels, the density of each band on the immunoblots was quantified using ImageJ. The optical density of striatal TH-positive fibers was determined with ImageJ. The data are presented as percentages of the values for the intact side, which was defined as 100%. Quantitative data are presented as the mean ± SEM. Representative morphological images were taken from at least three independent experiments with similar results. Imaging data were analyzed using the National Institutes of Health ImageJ software. The data were analyzed using the Statistical Package for the Social Sciences (SPSS) (version 11.0). Statistical significance was assessed using an unpaired two-tailed Student’s *t*-test or one or two ANOVA with Student–Newman–Keuls post hoc analysis. Spearman’s test was used for correlation analysis. Differences were considered significant at *P* < 0.05.

### In vitro kinase assay

Briefly, His_6_-tagged NEDD4-1 (1 μg) was incubated with 100 ng of the C-terminal kinase domain of PAK4 (aa 295–end) (a gift from Professor Soo-Jae Lee, Chungbuk National University College of Pharmacy) or GST in kinase assay buffer (20 mM Tris–HCl, pH 8.0; 10 mM MgCl_2_, and 1 mM dithiothreitol) containing 100 μM ATP and 5 μCi [γ-32P] ATP for 0.5 h at 30 °C. Reaction products were resolved by SDS-PAGE and transferred to PVDF membranes. Phosphorylated NEDD4-1 was detected by autoradiography.

### In vitro ubiquitination assay

In vitro ubiquitination assay was performed using an auto-ubiquitination kit (BML-UW0970, Enzo Life Science) following the manufacturer’s protocol. Briefly, for analysis of E3 ligase activity of NEDD4-1, NEDD4-1 was incubated with E1, E2, and ubiquitin in ubiquitination buffer at 37 °C for 1 h. The reaction was stopped with 5× SDS-PAGE sample buffer, and proteins were resolved by SDS-PAGE and transferred to PVDF membranes. Self-ubiquitinated NEDD4-1 was detected by anti-ubiquitin antibody.

## Supplementary information

Supplementary information

Original Data File

checklist

## Data Availability

All data generated during or analyzed during this study are included in this published article and its Supplementary Information files.
