# Supplementary material for: p21-activated kinase 4 controls the aggregation of α-synuclein by reducing the monomeric and aggregated forms of α-synuclein: involvement of the E3 ubiquitin ligase NEDD4-1
Source: Cell Death Dis. 2022 Jun 30;13(6):575. doi: 10.1038/s41419-022-05030-1 (PMC9247077; doi:10.1038/s41419-022-05030-1)
Supplement: Supplementary file 1 — Supplementary information [file 41419_2022_5030_MOESM1_ESM.docx]

**Supplementary information**

**p21-activated kinase 4 controls the aggregation of α-synuclein by reducing the monomeric and aggregated forms of α-synuclein:**

**involvement of the E3 ubiquitin ligase NEDD4-1**

So-Yoon Won, Jung-Jin Park, Soon-Tae You, Jong-A Hyeun, Hyong-Kyu Kim,

Byung Kwan Jin, Catriona McLean, Eun-Young Shin and Eung-Gook Kim^*^

**Supplemental Fig. 1**


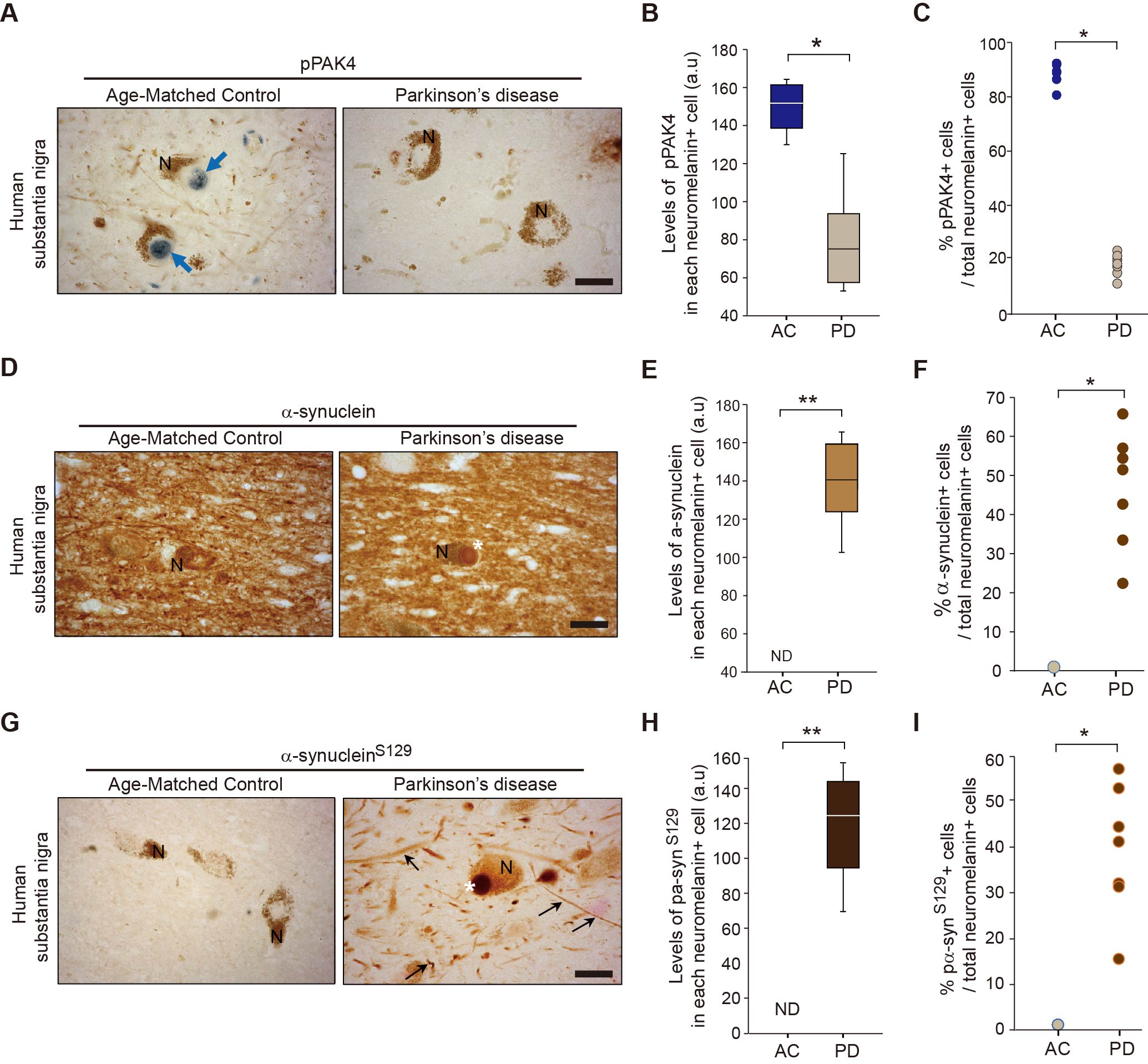


**Supplemental Fig. 1 Status of PAK4 activity and α-synuclein aggregates in age-matched controls versus patients with PD.** (**A**) Representative images of neuromelanin-positive dopamine neurons and pPAK4-positive cells (blue) in the SN of postmortem brains from age-matched controls (AC) and patients with PD. Arrows indicate pPAK4 staining (n = 7 for each group), Scale bar, 25 µm. (**B**) Quantification of the signal intensity for pPAK4 in neuromelanin-positive dopamine neurons (AC, n = 42; PD, n = 58). (**C**) Quantification of the percentage of pPAK4-positive cells in the SN of postmortem brains from age-matched controls and PD patients (AC, n = 42; PD, n = 58). (**D**) Representative images of neuromelanin-positive dopamine neurons and α-synuclein-positive cells (blue) in the SN of postmortem brains from age-matched controls and PD patients (n = 7 for each group). Scale bar, 25 µm. (**E**) Quantification of the signal intensity for α-synuclein in neuromelanin-positive dopamine neurons (AC, n = 50; PD, n = 57). (**F**) Quantification of the percentage of α-synuclein-positive cells in the SN of postmortem brains from age-matched controls and PD patients (AC, n = 50; PD, n = 57). (**G**) Representative images of neuromelanin-positive dopamine neurons and phosphorylated α-syn^S129^-positive cells (brown) in the SN of postmortem brains from age-matched controls (AC) and patients with PD. (n = 7 for each group) Scale bar, 25 µm. (**H**) Quantification of the signal intensity for pα-syn^S129^ in neuromelanin-positive dopamine neurons (AC, n = 50; PD, n = 64). (**I**) Quantification of the percentage of pα-syn^S129^-positive cells in the SN of postmortem brains from age-matched controls and PD patients (AC, n = 50; PD, n = 64). The data are presented as the mean ± SEM. **P* < 0.01, ***P* < 0.001. Unpaired Student’s t-test (b, c, e, f, h, i). N, neuromenlanin. *, Lewy body.

**Supplemental Fig. 2**


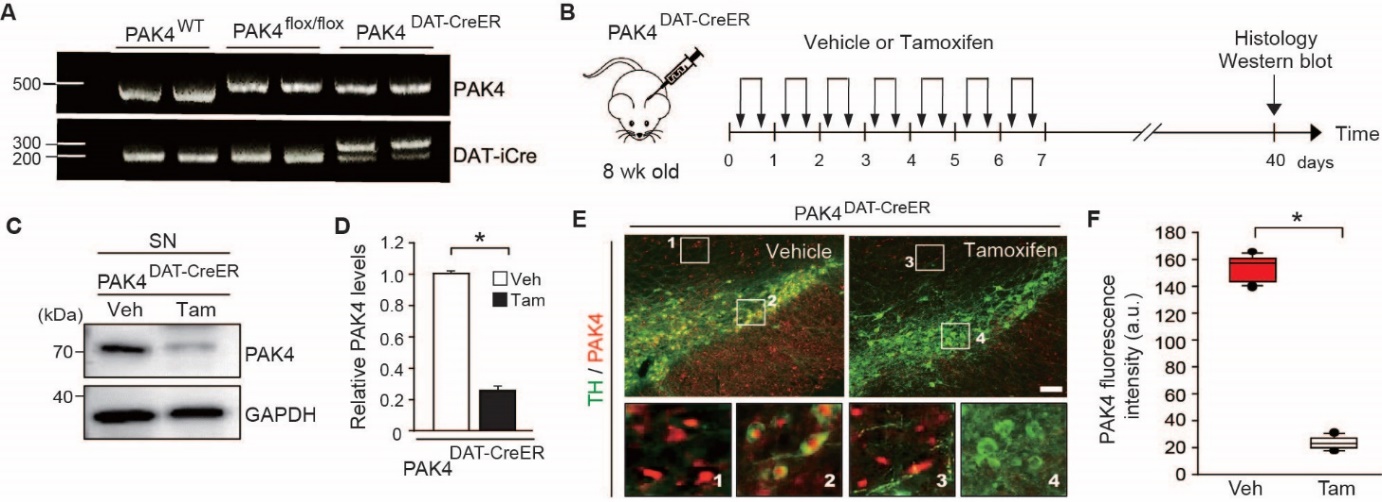


**Supplemental Fig. 2** Tamoxifen-induced PAK4 depletion in dopamine neurons of PAK4 DAT-**CreER mice.** (**A**) Representative PCR analysis to confirm the expected genotypes. (**B**) Experimental scheme. (**C**) Immunoblotting for PAK4 in the SN of PAK4DAT-CreER mice treated with vehicle (Veh) or tamoxifen (Tam). (**D**) Quantification of the blot in (C); PAK4 levels were normalized to GAPDH (n =3 for each group). (**E**) Double labeling for PAK4 and TH (green) in the SN from vehicle- or tamoxifen-injected mice [compare Box 2 (Vehicle) to Box 4 (Tamoxifen)]. (**F**) Quantification of PAK4 staining intensity in TH-positive dopamine neurons. (a/u., arbitrary units). The data are presented as the mean ± SEM. **P* < 0.001. Unpaired Student’s t-test

**Supplemental Fig. 3**


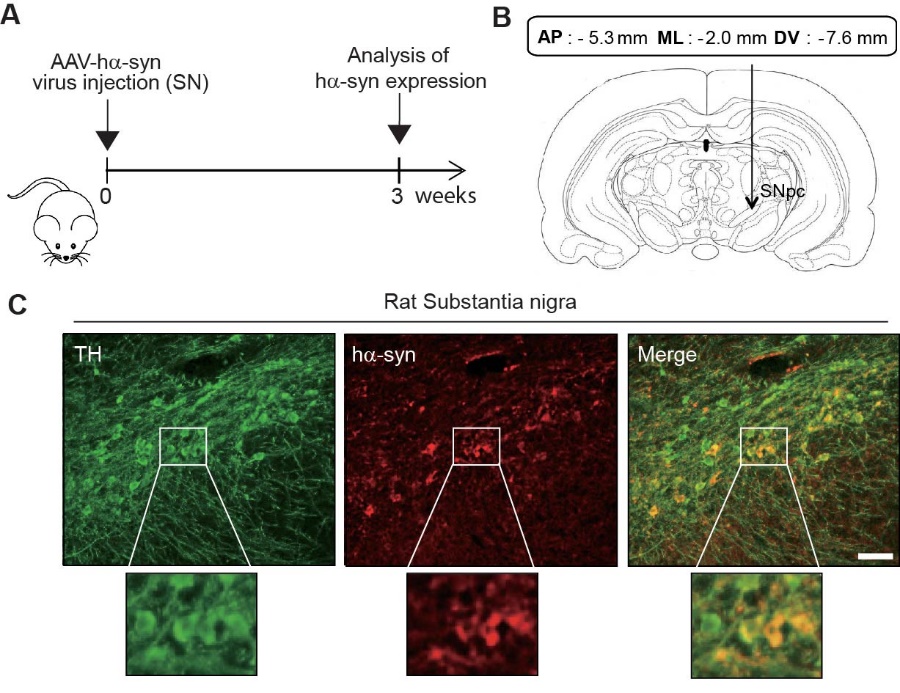


**Supplemental Fig. 3 Monitoring of AAV-mediated gene delivery into TH-positive dopamine neurons in SN.** (**A**) Experimental scheme. (**B**) Illustration showing stereotaxic intranigral injection of AAV–hα-synuclein. (**C**) The presence of expressed hα-synuclein in TH-positive (green) dopamine neurons 3 weeks after intranigral injection of AAV– hα-synuclein. Scale bar, 50 µm

**Supplemental Fig. 4**


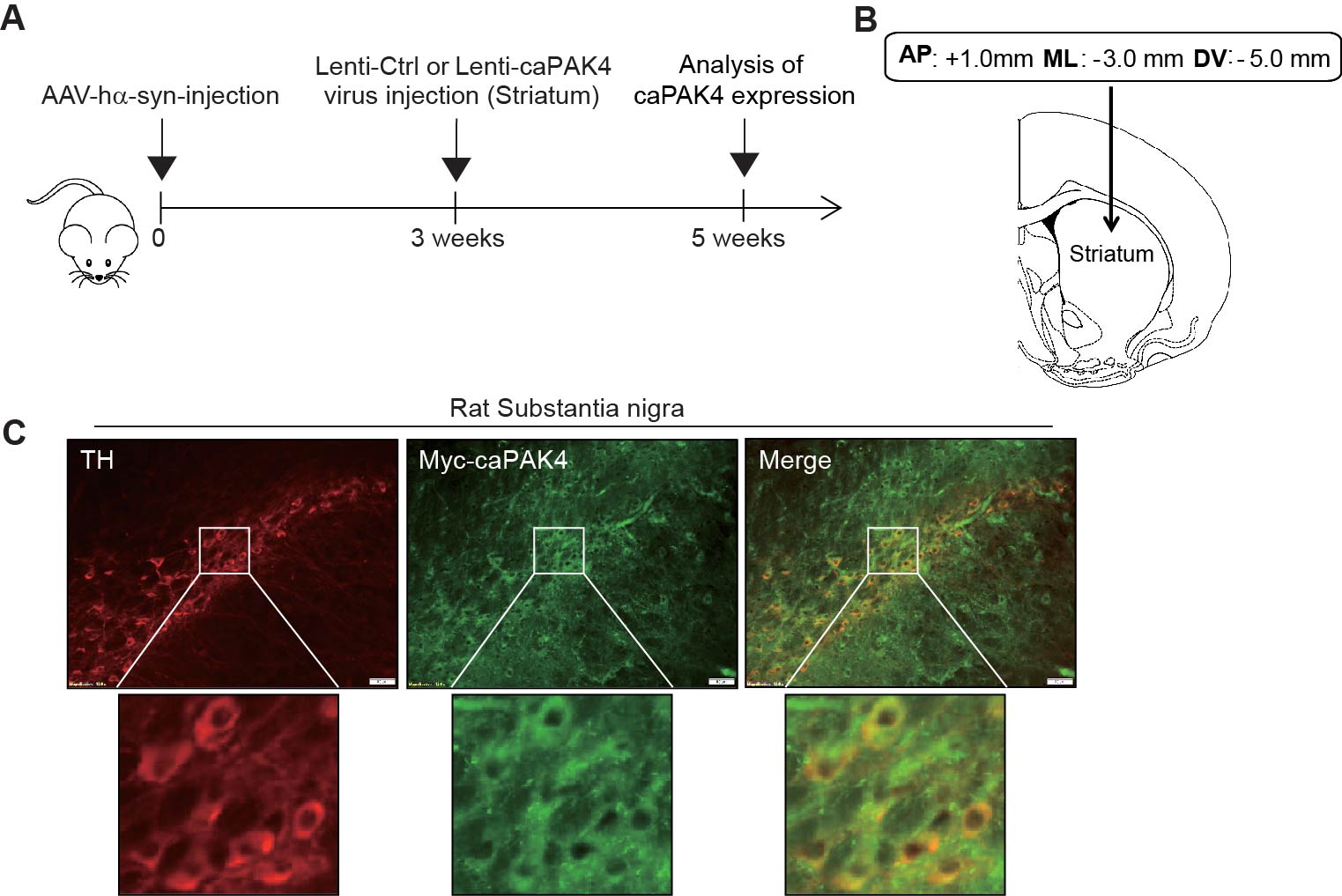


**Supplemental Fig. 4 Monitoring of lentivirus-mediated gene delivery into TH-positive dopamine neurons in SN.** (**A**) Experimental scheme. (**B**) Illustration showing stereotaxic intrastriatal injection of lenti-Ctrl/caPAK4. (**C**) Expression of caPAK4 (GFP; green) in TH-positive dopamine neurons 2 weeks after lentivirus injection. Scale bar, 50 µm

**Supplemental Fig. 5**


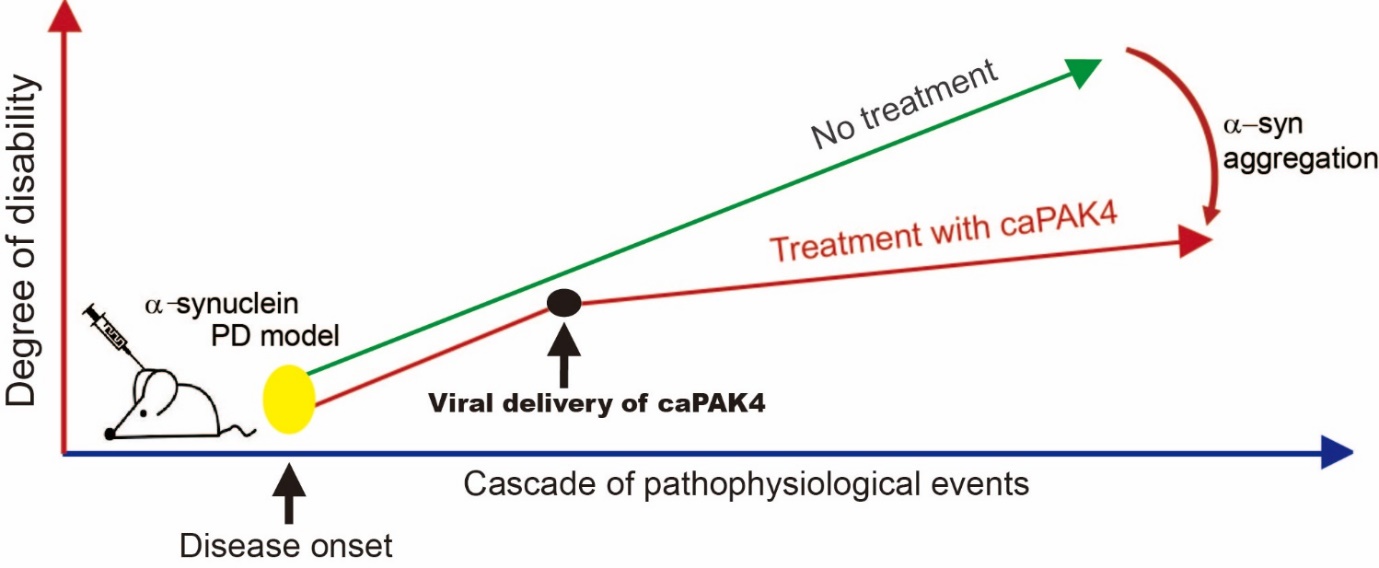


**Supplemental Fig. 5** Schematic representation of disease-modifying effect of caPAK by targeting alpha-synuclein aggregation.

**Supplemental Table S1**

| **Human post-mortem tissue used for immunohistochemistry.** | | | |
| --- | --- | --- | --- |
| **Age-matched control**  **(Case #)** | **Age (years)** | **Post-mortem interval**  **(hours)** | **Gender** |
| 04/250 | 79.6 | 31.5 | Male |
| 07/239 | 78.8 | 19 | Female |
| 04/034 | 73.7 | 26.5 | Female |
| 04/112 | 73.5 | 22 | Male |
| 06/144 | 69.4 | 24 | Male |
| 08/026 | 67.3 | 24 | Female |
| V11/052 | 63.6 | 54.5 | Male |
| **Parkinson’s disease**  **(Case #)** | **Age (years)** | **Post-mortem interval**  **(hours)** | **Gender** |
| V11/073 | 80.1 | 65 | Male |
| 07/566 | 78.4 | 31 | Male |
| 05/413 | 72.7 | 45 | Male |
| V11/042 | 72.1 | 25 | Male |
| 08/319 | 70 | 32.5 | Male |
| 09/260 | 66.8 | 20 | Female |
| 03/819 | 63.6 | 56 | Female |
| **Human post-mortem tissue used for immunoblotting.** | | | |
| **Age-matched control**  **(Case #)** | **Age (years)** | **Post-mortem interval**  **(hours)** | **Gender** |
| 04/250 | 79.6 | 31.5 | Male |
| 04/034 | 73.7 | 26.5 | Female |
| 04/112 | 73.5 | 22 | Male |
| 06/144 | 69.4 | 24 | Male |
| **Parkinson’s disease**  **(Case #)** | **Age (years)** | **Post-mortem interval**  **(hours)** | **Gender** |
| V11/073 | 80.1 | 65 | Male |
| 05/413 | 72.7 | 45 | Male |
| V11/042 | 72.1 | 25 | Male |
| 08/319 | 70 | 32.5 | Male |
