# Supplementary material for: p21-activated kinase 4 controls the aggregation of α-synuclein by reducing the monomeric and aggregated forms of α-synuclein: involvement of the E3 ubiquitin ligase NEDD4-1
Source: Cell Death Dis. 2022 Jun 30;13(6):575. doi: 10.1038/s41419-022-05030-1 (PMC9247077; doi:10.1038/s41419-022-05030-1)

**Supplemental Material**

**p21-activated kinase 4 controls the aggregation of α-synuclein by reducing the monomeric and aggregated forms of α-synuclein:**

**involvement of the E3 ubiquitin ligase NEDD4-1**


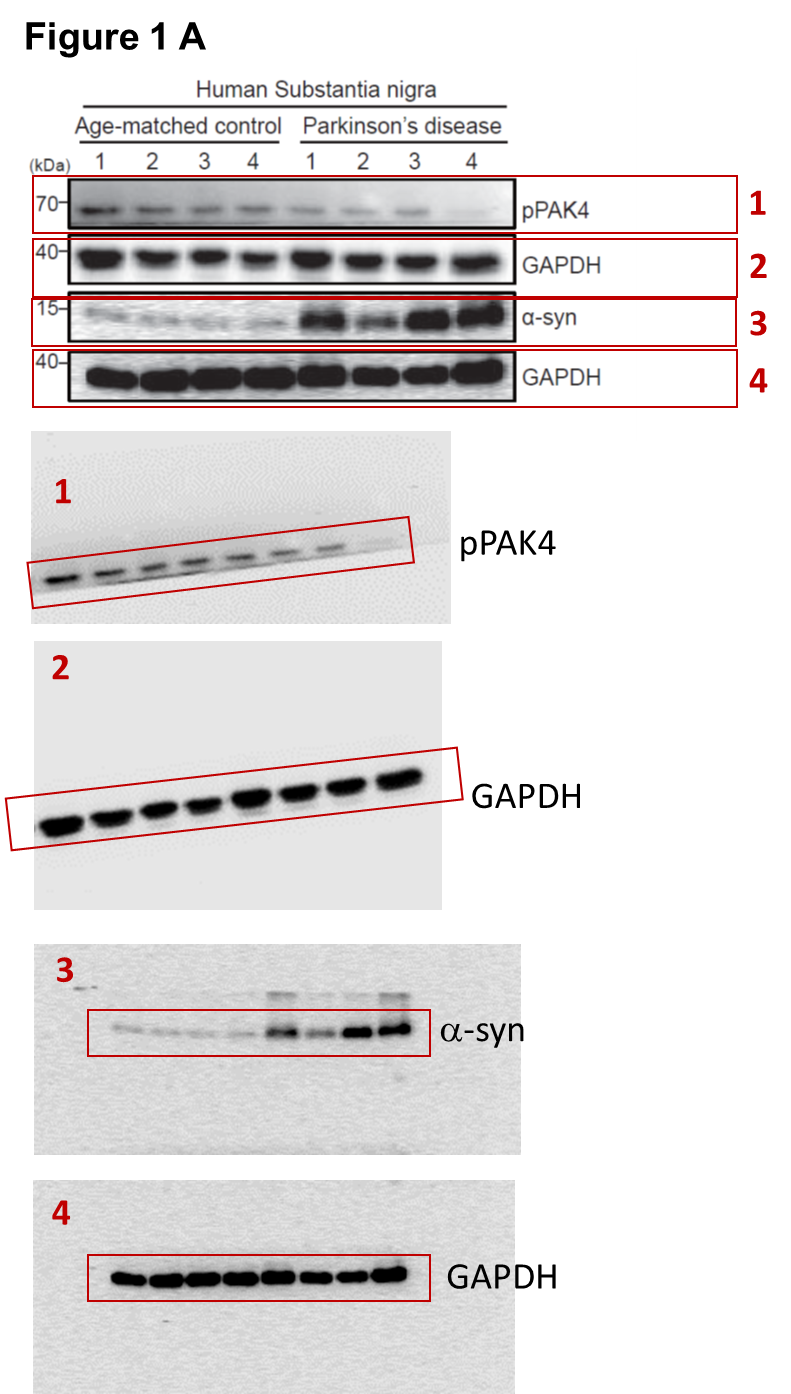


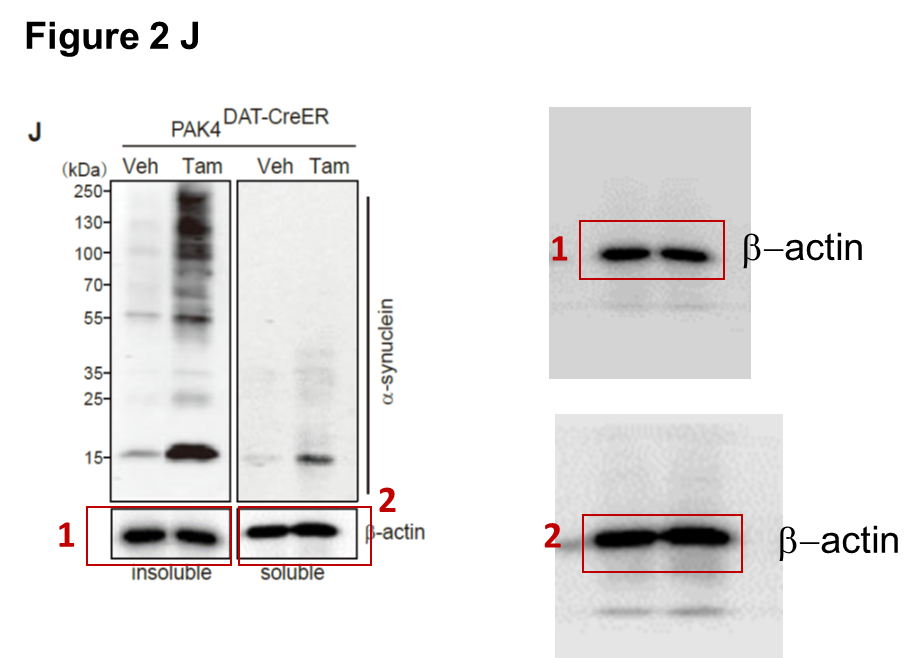


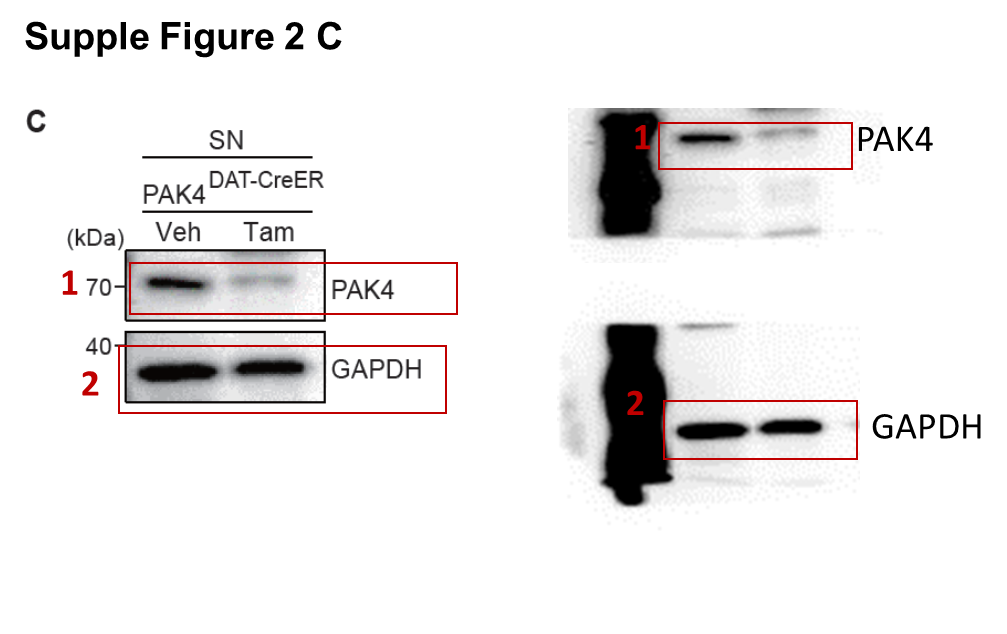


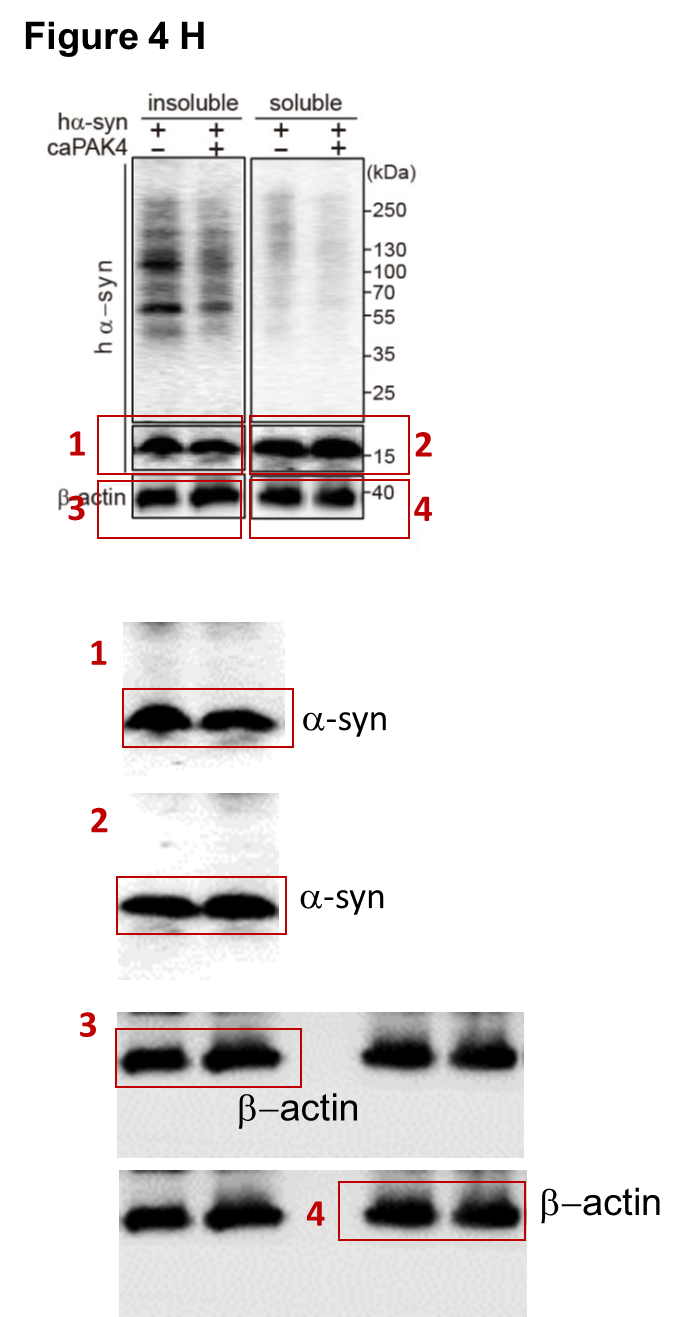


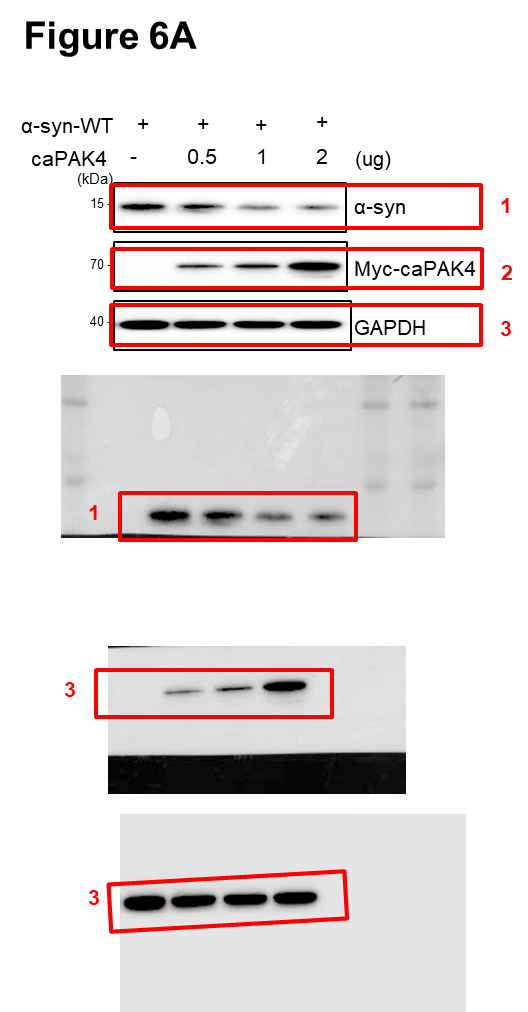


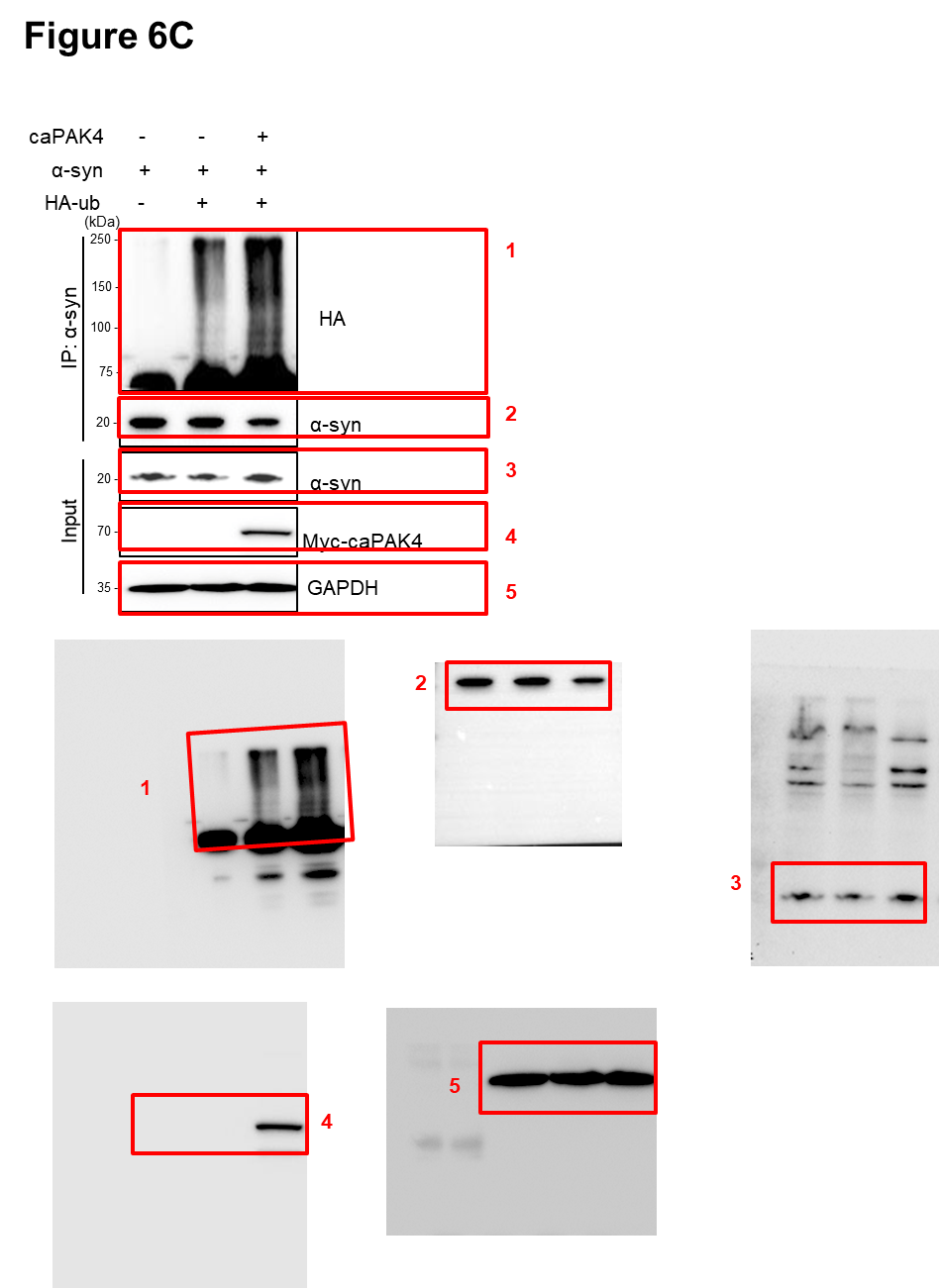


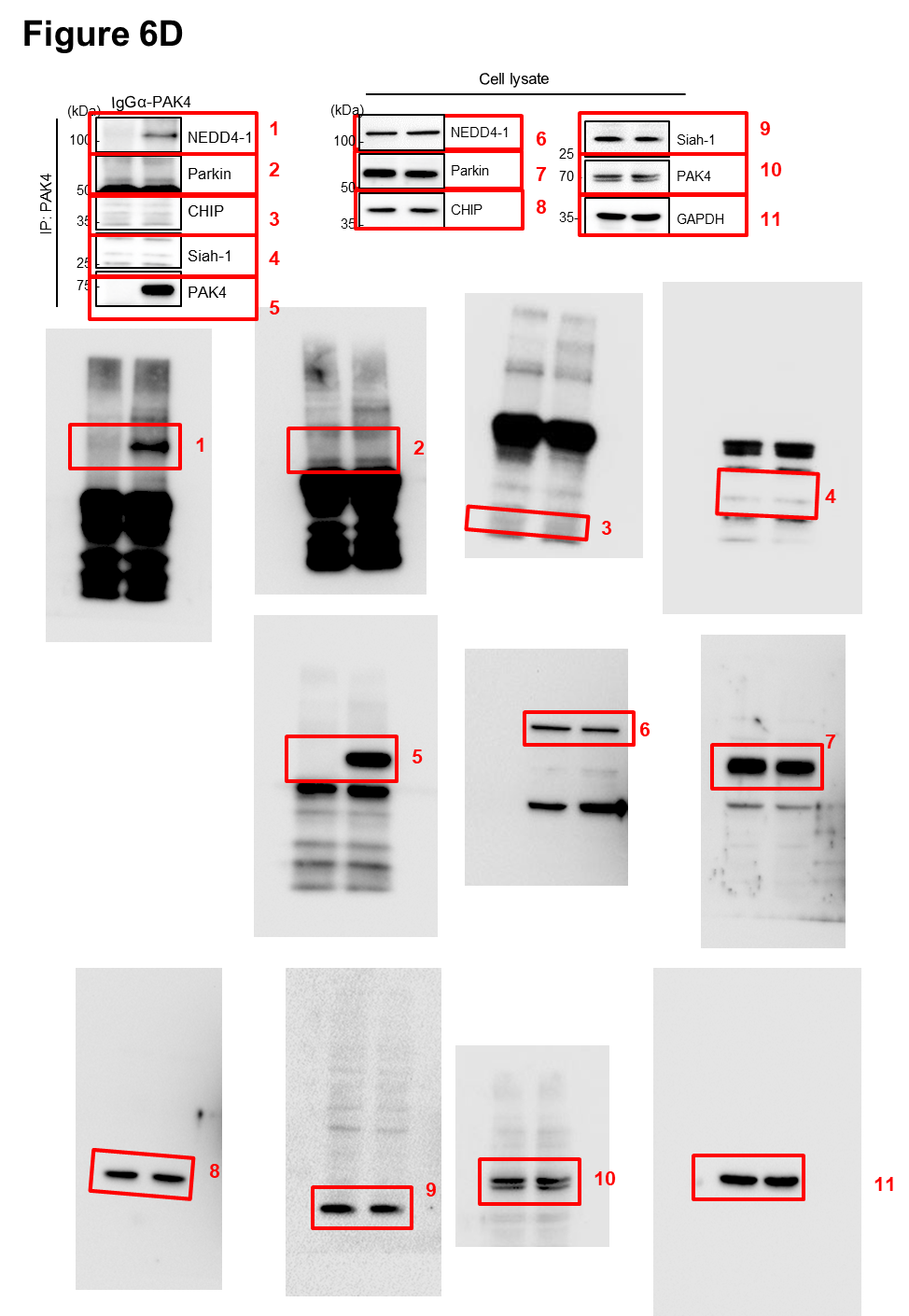


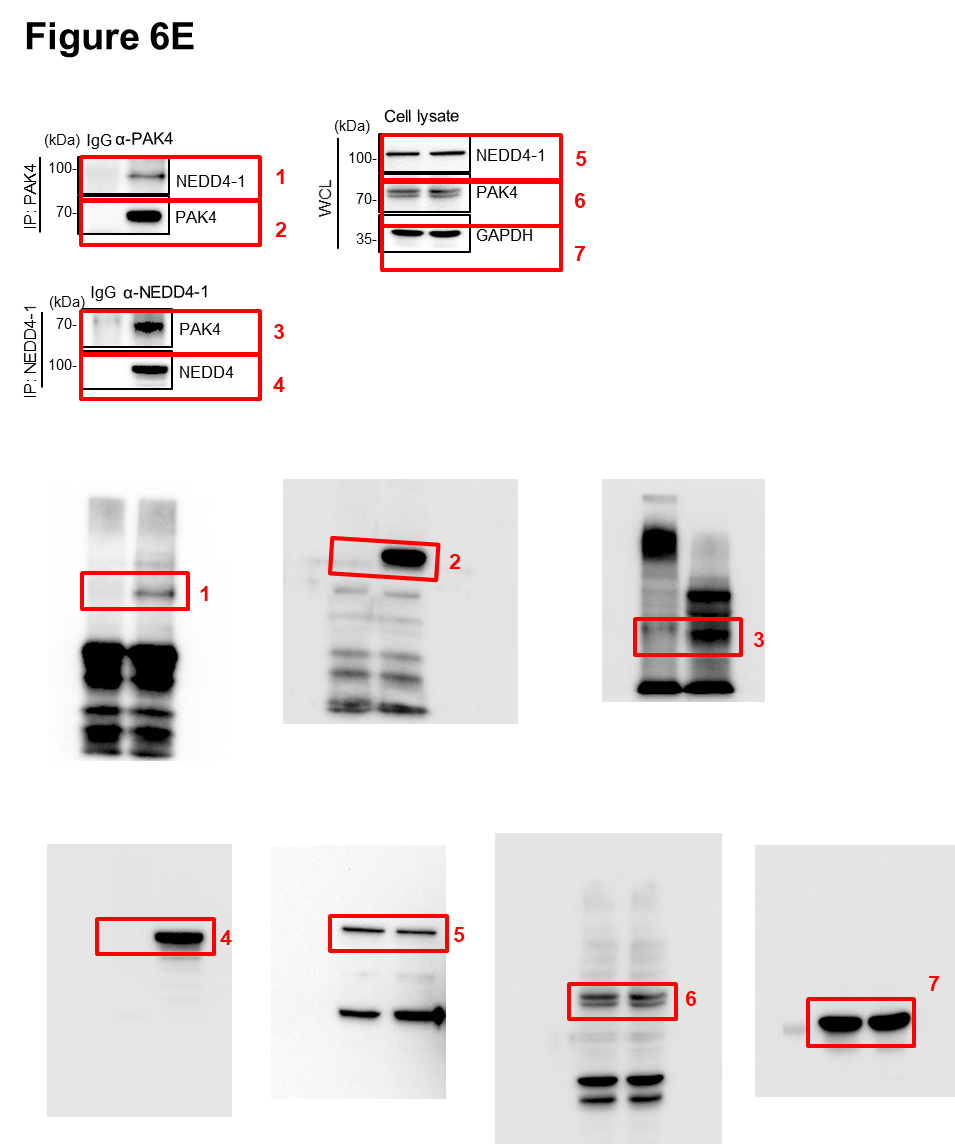


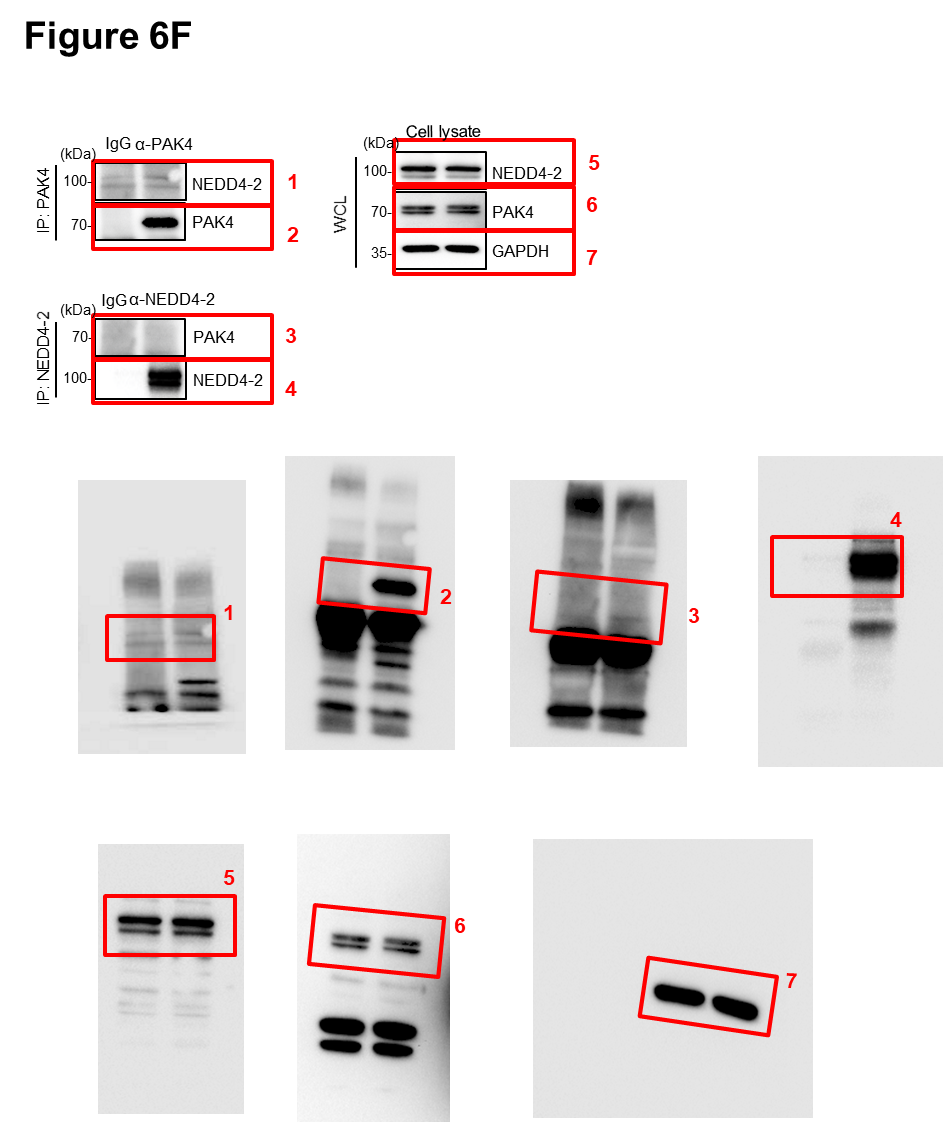


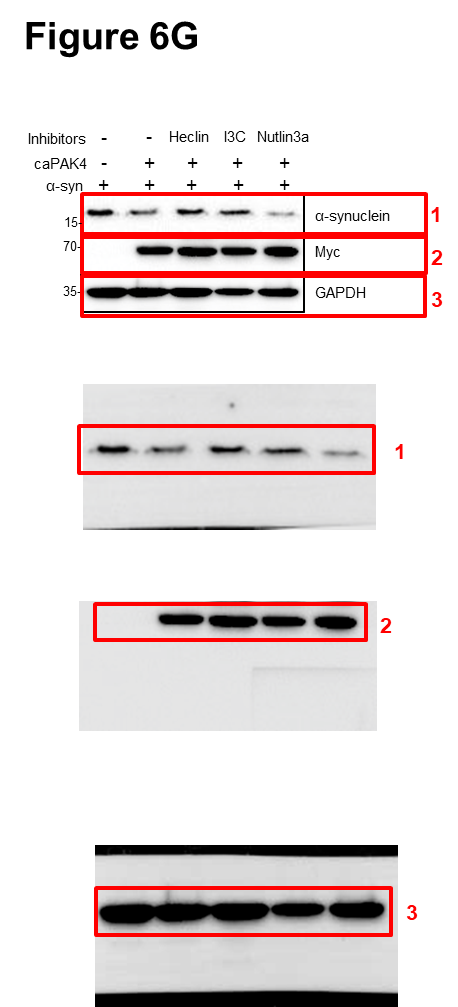


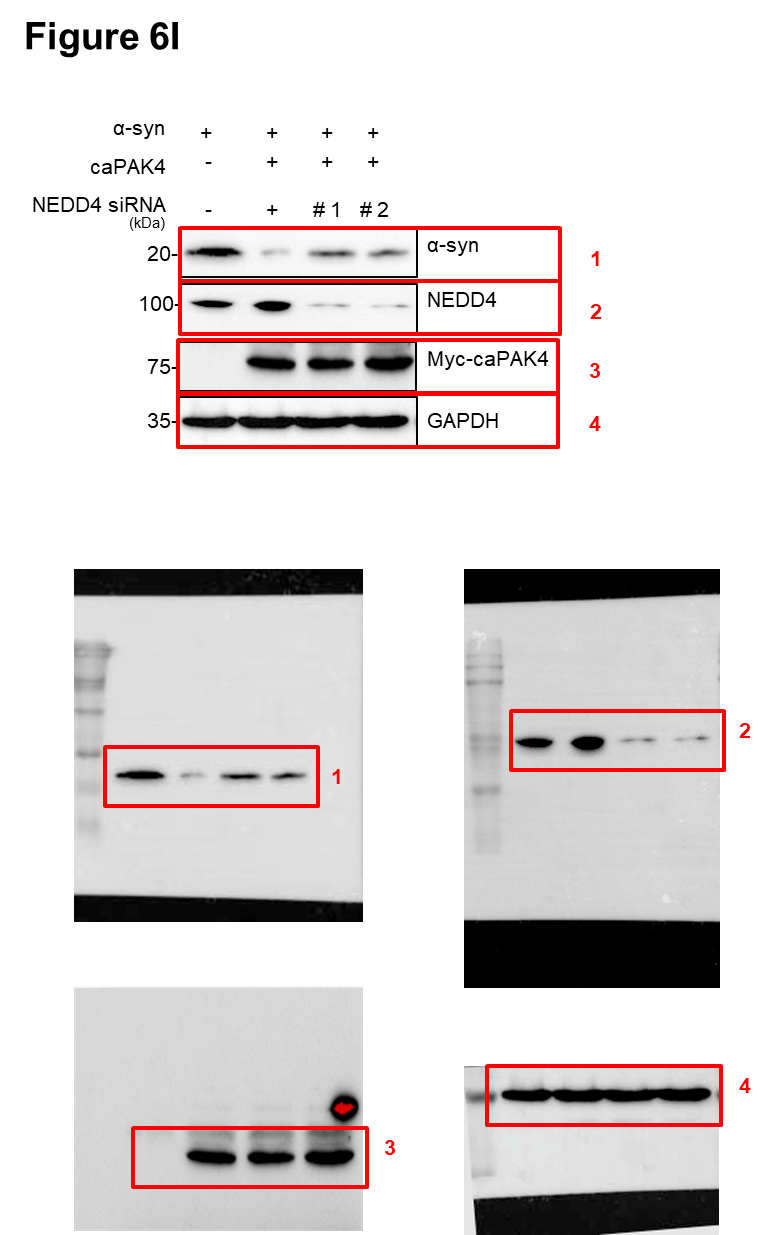


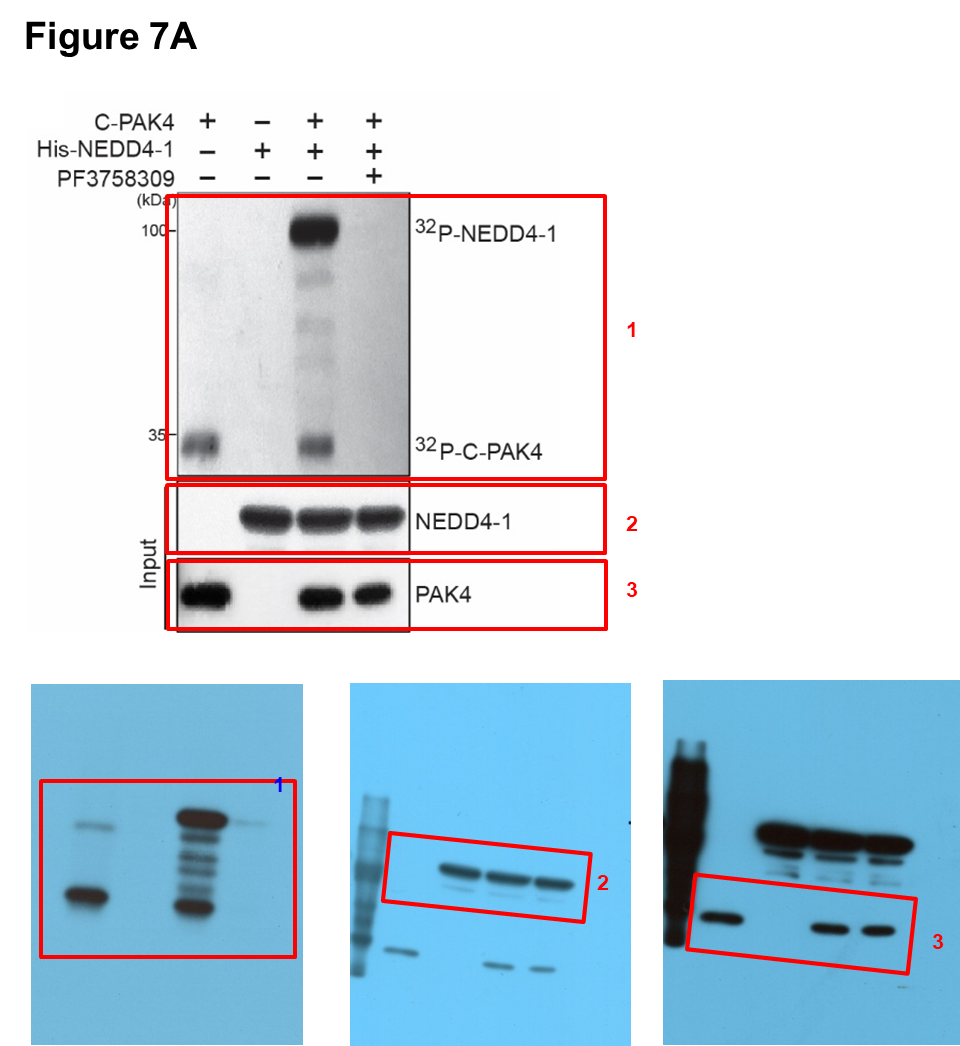


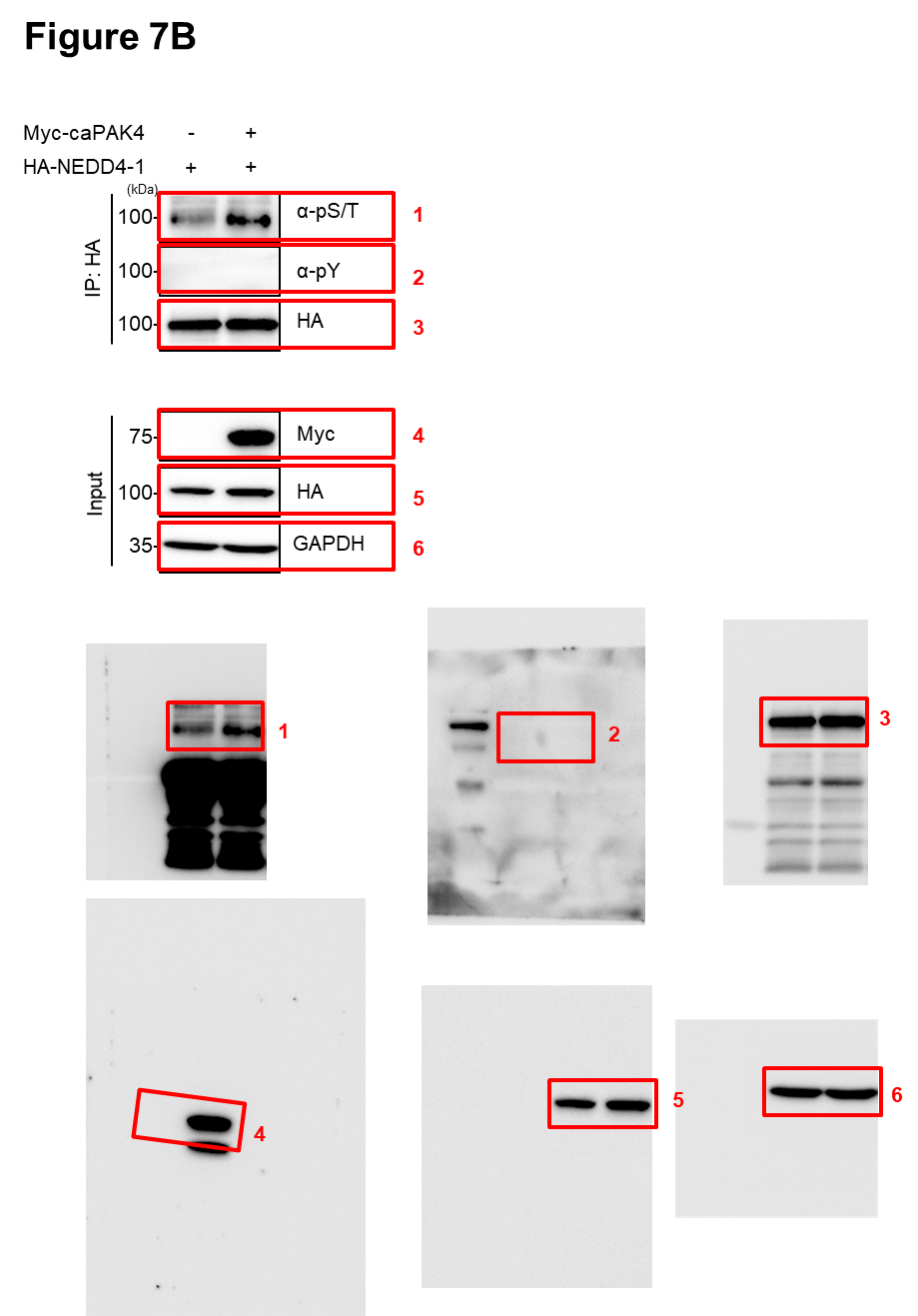


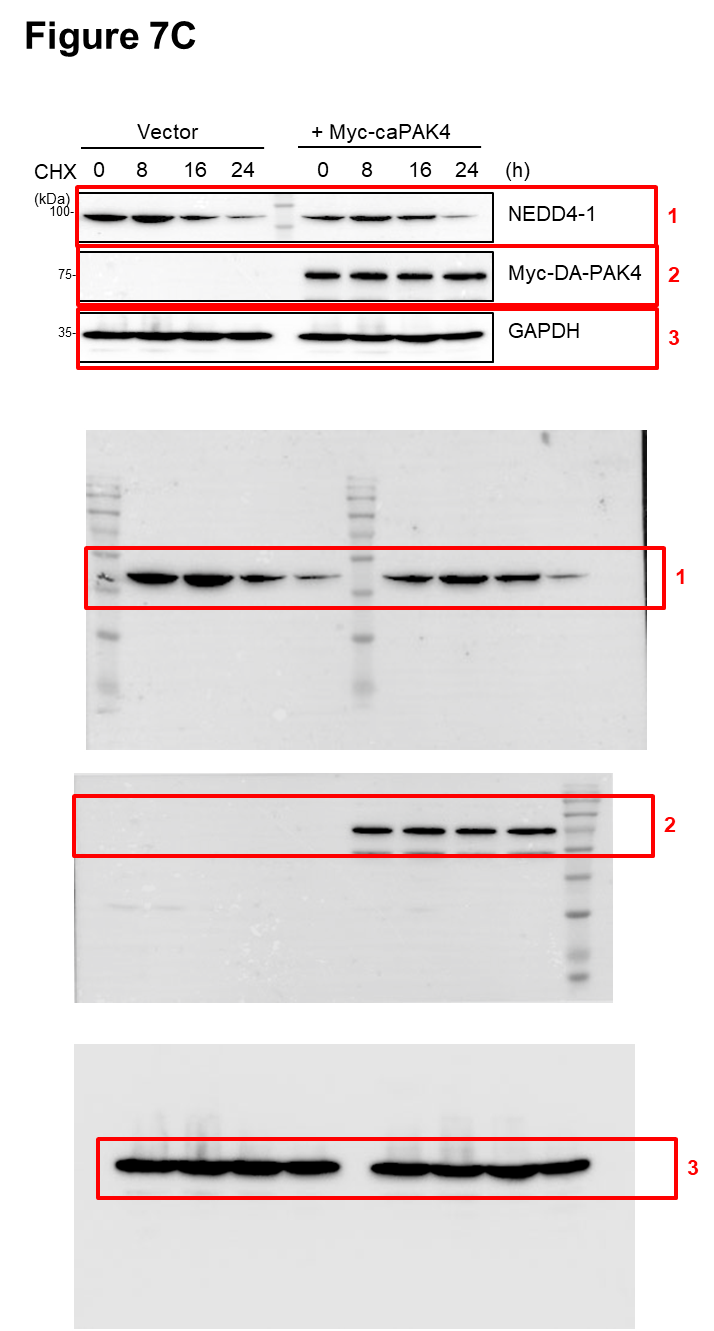


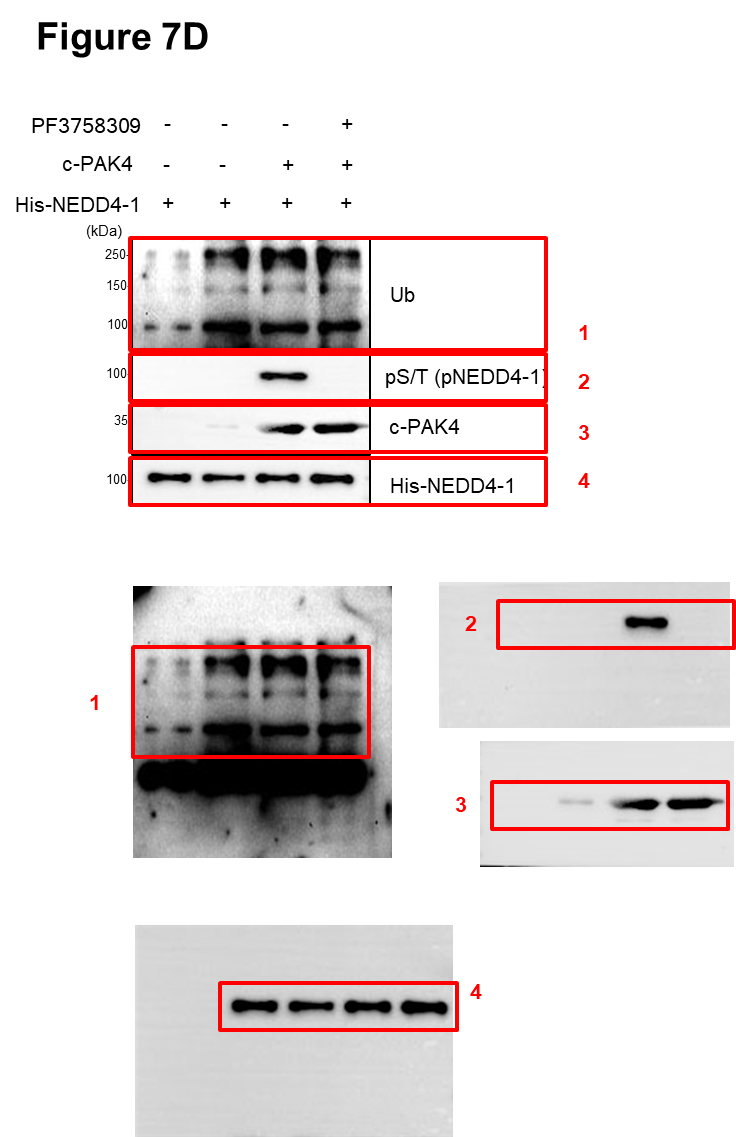


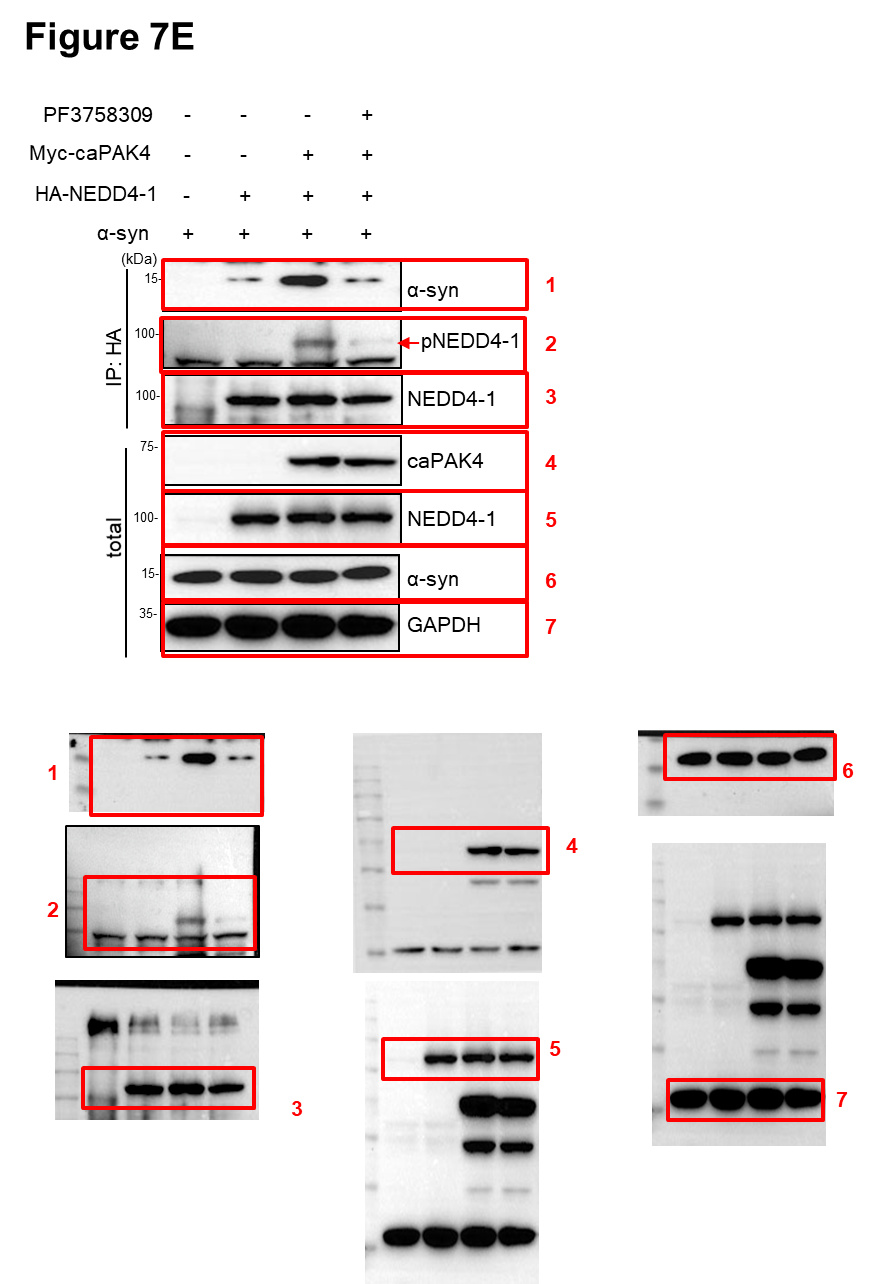

Supplement: Supplementary file 2 — Original Data File [file 41419_2022_5030_MOESM2_ESM.docx]
